# Circularly Polarized Luminescence from New Heteroleptic Eu(III) and Tb(III) Complexes

**DOI:** 10.1021/acs.inorgchem.3c00196

**Published:** 2023-06-01

**Authors:** Silvia Ruggieri, Silvia Mizzoni, Chiara Nardon, Enrico Cavalli, Cristina Sissa, Michele Anselmi, Pier Giorgio Cozzi, Andrea Gualandi, Martina Sanadar, Andrea Melchior, Francesco Zinna, Oliver G. Willis, Lorenzo Di Bari, Fabio Piccinelli

**Affiliations:** † Luminescent Materials Laboratory, DB, University of Verona, and INSTM, UdR Verona, Strada Le Grazie 15, 37134 Verona, Italy; ‡ Department of Chemistry, Life Sciences and Environmental Sustainability, Parma University, Parco Area delle Scienze, 17/a, 43124 Parma, Italy; § Department of Chemistry “G. Ciamician”, University of Bologna, via Selmi 2, 40126 Bologna, Italy; ∥ Center for Chemical Catalysis - C3, Alma Mater Studiorum - Università di Bologna, Via Selmi 2, 40126 Bologna, Italy; ⊥ Dipartimento Politecnico di Ingegneria e Architettura, Laboratorio di Tecnologie Chimiche, Università di Udine, via Cotonificio 108, 33100 Udine, Italy; # Department of Chemistry and Industrial Chemistry, University of Pisa, via Moruzzi 13, 56124 Pisa, Italy

## Abstract

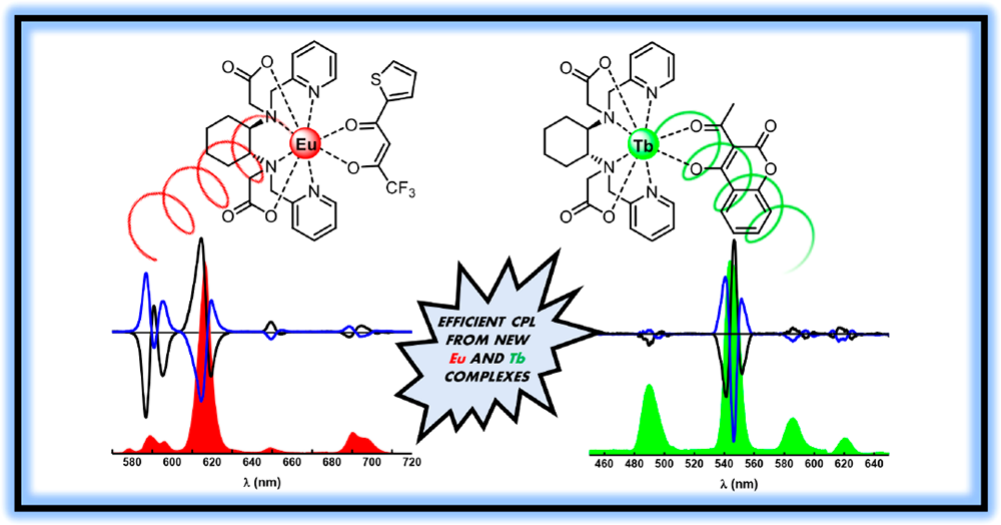

The complexes [Eu(bpcd)(tta)], [Eu(bpcd)(Coum)], and [Tb(bpcd)(Coum)] [tta = 2-thenoyltrifluoroacetyl-acetonate, Coum = 3-acetyl-4-hydroxy-coumarin, and bpcd = *N*,*N*′-bis(2-pyridylmethyl)-*trans*-1,2-diaminocyclohexane-*N*,*N*′-diacetate] have been synthesized and characterized from photophysical and thermodynamic points of view. The optical and chiroptical properties of these complexes, such as the total luminescence, decay curves of the Ln(III) luminescence, electronic circular dichroism, and circularly polarized luminescence, have been investigated. Interestingly, the number of coordinated solvent (methanol) molecules is sensitive to the nature of the metal ion. This number, estimated by spectroscopy, is >1 for Eu(III)-based complexes and <1 for Tb(III)-based complexes. A possible explanation for this behavior is provided via the study of the minimum energy structure obtained by density functional theory (DFT) calculations on the model complexes of the diamagnetic Y(III) and La(III) counterparts [Y(bpcd)(tta)], [Y(bpcd)(Coum)], and [La(bpcd)(Coum)]. By time-dependent DFT calculations, estimation of donor–acceptor (D–A) distances and of the energy position of the S_1_ and T_1_ ligand excited states involved in the *antenna* effect was possible. These data are useful for rationalizing the different sensitization efficiencies (η_sens_) of the *antennae* toward Eu(III) and Tb(III). The tta ligand is an optimal *antenna* for sensitizing Eu(III) luminescence, while the Coum ligand sensitizes better Tb(III) luminescence {ϕ_ovl_ = 55%; η_sens_ ≥ 55% for the [Tb(bpcd)(Coum)] complex}. Finally, for the [Eu(bpcd)(tta)] complex, a sizable value of *g*_lum_ (0.26) and a good quantum yield (26%) were measured.

## Abstract 2

Chiral, DACH-based Eu(III) and Tb(III) heteroleptic complexes exhibit good metal-centered circularly polarized luminescence activity upon excitation of the *antenna* ligands. The tta and Coum ligands are optimal *antennae* for Eu(III) and Tb(III) luminescence, respectively. The Tb(III)-based complex shows a very good overall quantum yield of ∼55%.

## Introduction

**Figure 1:**
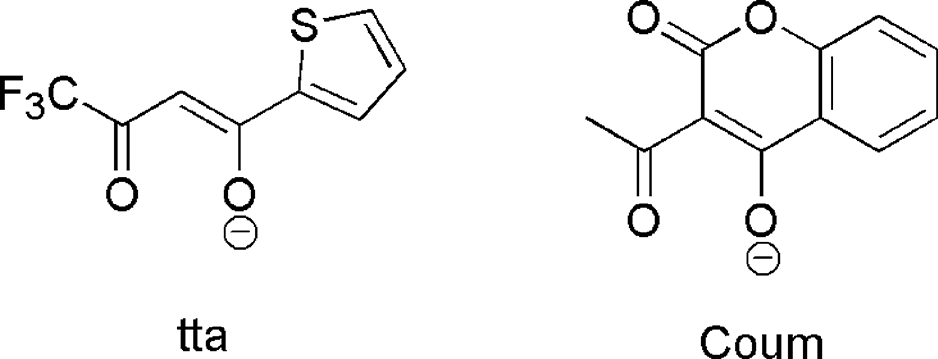
Ligands employed in this work.

Circularly polarized luminescence (CPL) is a chiroptical phenomenon that is attracting more interest in materials chemistry and physics thanks to the broad range of possible biological^1−5^ and technological applications,^6−15^ as in the case of the design of organic light-emitting diodes (OLEDs) emitting circularly polarized (CP) light.^16−18^ Focusing our attention on these latter devices, we can find a logical application in displays in which the emitted polarized light is exploited to prevent reflection of ambient light and obtain high-contrast three-dimensional images and true black.^19^ In addition, a CP screen reduces the perceived distortion found at some angles when the display is viewed through a linearly polarized (LP) filter, significantly improving the outdoor viewing of laptops or smartphones. In this scenario, as the emission of CP light by trivalent lanthanide ions is a quite efficient phenomenon, luminescent lanthanide complexes play a pivotal role.^18^ The new quantity recently proposed by some of us^20^ and known as CPL brightness (*B*_CPL_) can be considered as useful tool for the design of efficient CPL phosphors for specific chiroptical applications, such as in CPL microscopy^21^ and possibly in CPL security inks.^7,22^ Moreover, a high *B*_CPL_ is correlated with a higher signal available for a CPL measurement.^23,24^*B*_CPL_ takes into account the absorption extinction coefficient and quantum yield along with the *g*_lum_ factor and, for a selected lanthanide transition, is defined as *B*_CPL_ = β_i_ε_λ_ϕ_ovl_|*g*_lum_|/2 = β_i_*B*|*g*_lum_|/2, where β_i_ is the so-called branching ratio (β; 0 ≤ β ≤ 1), ε_λ_ is the molar extinction coefficient, ϕ_ovl_ is the overall quantum yield,^25^ and *g*_lum_(26) is the dissymmetry factor associated with the considered transition β_i_ = *I*_i_/∑_*j*_*I*_*j*_, where *I*_i_ is the integrated intensity of the considered transition and ∑_*j*_*I*_*j*_ is the summation of the integrated intensities over all of the transitions. In the case of lanthanide complexes, optimal *B*_CPL_ values can be obtained in the presence of strong absorbing chromophores (high ε_λ_ values), good ligand-to-metal energy transfer (LMET or *antenna* effect), and luminescence efficiency from Ln(III) (both contributing to high ϕ_ovl_ values)_._ Finally, the emitted light should be strongly polarized, with the difference in the intensity between the right- and left-handed components of the CP light being as large as possible. A promising strategy for obtaining a Ln(III)-based luminescent complex exhibiting sizable *B*_CPL_ values is to design heteroleptic complexes in which two different ligands are bound to the metal ion. One ligand takes care of efficiently sensitizing the Ln(III) luminescence, while the other one can trigger the required CPL activity, by virtue of the fact that it is chiral and nonracemic. As for the *antenna* that can sensitize Eu(III) and Tb(III) luminescence, the anion derived from 3-acetyl-4-hydroxycoumarin (Coum)^27^ has been selected for both ions, while 2-thenoyltrifluoroacetyl-acetonate (tta) has been selected as the ligand for only Eu(III) (Figure 1). It is well-known that the latter ligand efficiently sensitizes the luminescence of the Eu(III) ion by virtue of the small energy gap between the accepting levels of the europium ion and the triplet state of the ligand and the increased anisotropy around the metal ion.^28^

**Figure 2:**
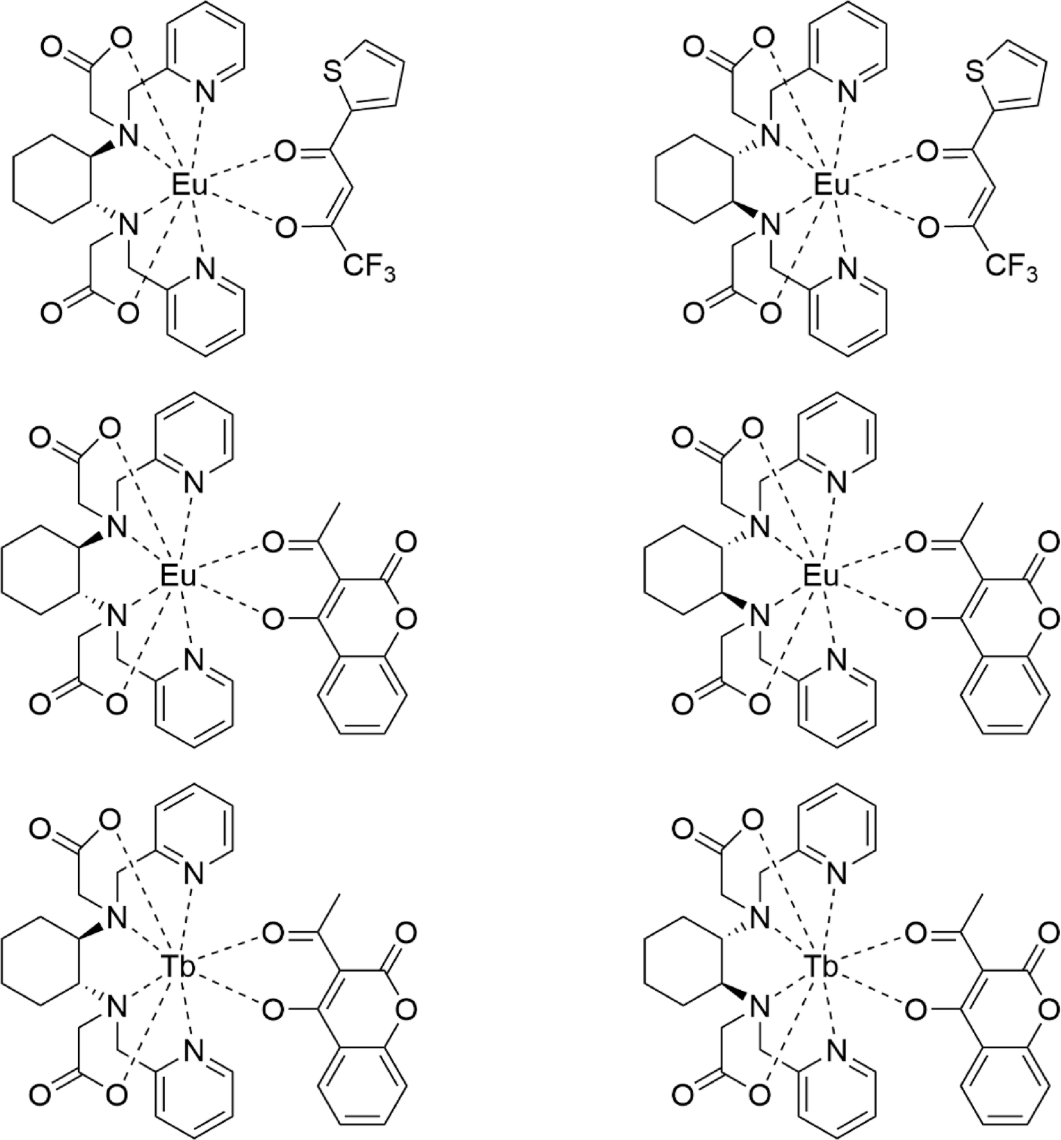
Complexes investigated in this work: (top) (*R,R*)-[Eu(bpcd)(tta)] (left) and (*S,S*)-[Eu(bpcd)(tta)] (right), (middle) (*R,R*)-[Eu(bpcd)(Coum)] (left) and (*S,S*)-[Eu(bpcd)(Coum)] (right), and (bottom) (*R,R*)-[Tb(bpcd)(Coum)] (left) and (*S,S*)-[Tb(bpcd)(Coum)] (right).

As the chiral bpcd ligand can induce a significant chiroptical response stemming from Eu(III) and Tb(III) ions,^29^ we decided to employ this ligand to produce CPL activity from our complexes. Therefore, in this work, we synthesized and spectroscopically characterized three different complexes (in both enantiomeric forms), namely, [Eu(bpcd)(tta)], [Eu(bpcd)(Coum)], and [Tb(bpcd)(Coum)]; the structures are shown in Figure 2.

Also, an ultraviolet–visible (UV–vis) titration study and density functional theory (DFT) calculations were carried out to characterize the stability and structural features of the complexes present in solution. Also, by means of time-dependent DFT (TD-DFT) calculations, the energies of the tta and Coum excited states involved in the energy transfer mechanism were determined, and a possible explanation for the different sensitization efficiencies of the different complexes has been provided.

## Experimental Section

EuCl_3_·6H_2_O and TbCl_3_·6H_2_O (Aldrich, 98%) and 2-thenoyltrifluoroacetyl-acetone (Htta, Alfa Aesar, 98%) were stored under vacuum for several days at 80 °C and then transferred to a glovebox.

Both enantiomers of the *N*,*N*′-bis(2-pyridylmethyl)-*trans*-1,2-diaminocyclohexane-*N*,*N*′-diacetic acid (H_2_bpcd) ligand, in the form of trifluoroacetate salt, were synthesized as previously reported in the literature.^30^

### Elemental Analysis

Elemental analyses were carried out by using an EACE 1110 CHNO analyzer.

### ESI-MS

Electrospray ionization mass spectra (ESI-MS) were recorded with a Finnigan LXQ Linear Ion Trap (Thermo Scientific, San Jose, CA) operating in positive ion mode. The data were acquired under the control of Xcalibur software (Thermo Scientific). A methanol solution of the sample was properly diluted and infused into the ion source at a flow rate of 10 μL/min with the aid of a syringe pump. The typical source conditions were as follows: transfer line capillary at 275 °C; ion spray voltage of 4.70 kV; and sheath, auxiliary, and sweep gas (N_2_) flow rates of 10, 5, and 0 arbitrary units, respectively. Helium was used as the collision damping gas in the ion trap set at a pressure of 1 mTorr.

The Coum ligand precursor was synthesized by modifying the procedure previously reported in the literature.^27^ In a 50 mL round-bottom flask equipped with a condenser under a nitrogen atmosphere, 4-hydroxy coumarine (839 mg, 5.18 mmol), dry and freshly distilled pyridine (10 mL), and 4-(dimethylamino)pyridine (DMAP, 32 mg, 0.26 mmol) were added. To this stirred suspension was added acetic anhydride (0.57 mL, 529 mg, 5.18 mmol), and the mixture was stirred at 50 °C for 24 h. Pyridine was evaporated under reduced pressure; the residue was dissolved in ethyl acetate (AcOEt, 20 mL), and 1 M aqueous HCl was added until the pH reached 1. The water phase was extracted with AcOEt (3 × 15 mL); the combined organic layers were dried over anhydrous Na_2_SO_4_, and the solvent was removed under reduced pressure. The crude was subjected to flash column chromatography (SiO_2_; 85:15 cyclohexane/AcOEt) to afford 3-acetyl-4-hydroxy-2*H*-chromen-2-one in 64% yield (673 mg, 3.3 mmol). Spectroscopic data (^1^H NMR and ^13^C NMR in Figures S11 and S12) are in agreement with those reported in the literature.^27^

**Figure 3:**
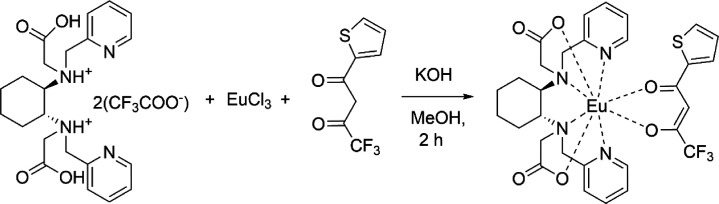
Synthesis of [Eu(bpcd)(tta)] complexes (the *R,R* enantiomer is chosen as a representative).

[Eu(bpcd)(tta)] (yield of 94%) was synthesized as follows (Figure 3). At room temperature, KOH (60 mg, 1.07 mmol) was added to a methanol (8 mL) solution of the desired enantiomer (*S,S* or *R,R*) of H_2_bpcd (200 mg, 0.31 mmol, as trifluoroacetate salt). After 15 min, EuCl_3_·6H_2_O (116 mg, 0.31 mmol) was added to form the first complex, [Eu(bpcd)]Cl. In another flask, KOH (21 mg, 0.37 mmol) was added to a methanol (4 mL) solution of Htta (2-thenoyltrifluoroacetyl-acetone, 68 mg, 0.31 mmol). This mixture was slowly added to the previously prepared solution containing the first complex, and the final mixture was stirred for 1 h. Then, the solvent was removed under reduced pressure, and the desired product was obtained as a yellow powder upon extraction in dichloromethane (8 × 3 mL) followed by solvent removal in vacuo.

Elemental analysis calcd for [Eu(bpcd)(tta)], C_30_H_30_EuF_3_N_4_O_6_S (MW 783.6): C, 45.98; H, 3.86; N, 7.15; O, 12.25. Found: C, 45.11; H, 3.80; N, 6.99; O, 12.37 (*R,R* isomer). Found: C, 45.28; H, 3.78; N, 7.07; O, 12.12 (*S,S* isomer). ESI-MS (scan ES+, *m*/*z*): 807.09 (100%), 805.09 (90%), 808.10 (35%) ([Eu(bpcd)(tta)] + Na)^+^; 784.09 (100%), 785.11 (30%), 783.11 (27%), 786.12 (10%) ([Eu(bpcd)(tta)] + H)^+^. ESI-MS (scan ES–, *m*/*z*): 220.99 (100%), 221.99 (10%) [tta]^−^.

**Figure 4:**
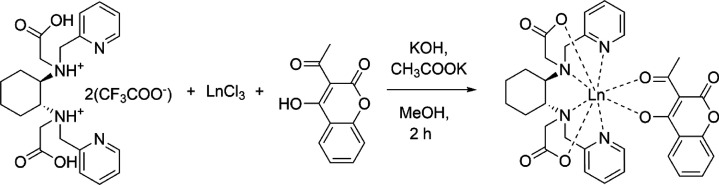
Synthesis of [Ln(bpcd)(Coum)] complexes (Ln = Eu or Tb). The *R,R* enantiomers are chosen as a representative.

[Eu(bpcd)(Coum)] and [Tb(bpcd)(Coum)] (yield of 95%) were synthesized as follows (Figure 4). At room temperature, EuCl_3_·6H_2_O (113 mg, 0.31 mmol) or TbCl_3_·6H_2_O (115.7 mg, 0.31 mmol) was added to a previously prepared methanol solution containing KOH (60 mg, 1.07 mmol) and H_2_bpcd (*S,S* or *R,R*) (200 mg, 0.31 mmol, as trifluoroacetate salt). In another flask, 3-acetyl-4-hydroxy-2*H*-chromen-2-one (63.3 mg, 0.31 mmol) was solubilized in methanol and added to a solution of NaOMe (16.7 mg, 0.31 mmol), in the same solvent (10 mL), with some drops of acetonitrile to improve its solubility. The deprotonated Coum ligand was slowly added to the solution containing the Ln(III) complex. The final mixture was stirred for 2 h. Then, the solvent was removed under reduced pressure, and the desired product was obtained as a yellow powder upon extraction in dichloromethane (8 × 3 mL) followed by solvent removal in vacuo.

Elemental analysis calcd for [Eu(bpcd)(Coum)](CH_3_OH), C_34_H_37_EuN_4_O_9_ (MW 797.64): C, 51.20; H, 4.68; N, 7.02; O, 18.05. Found: C, 51.22; H, 4.54; N, 7.11; O, 17.99 (*R,R* isomer). Found: C, 51.12; H, 4.60; N, 6.95; O, 17.91 (*S,S* isomer). ESI-MS (scan ES+, *m*/*z*): 789.14 (100%), 787.14 (90%), 790.14 (35%) ([Eu(bpcd)(Coum)] + Na)^+^.

Elemental analysis calcd for [Tb(bpcd)(Coum)], C_33_H_33_N_4_O_8_Tb (MW 772.56): C, 51.30; H, 4.31; N, 7.25; O, 16.57. Found: C, 51.25; H, 4.22; N, 7.18; O, 16.12 (*R,R* isomer). Found: C, 51.19; H, 4.12; N, 7.19; O, 16.33 (*S,S* isomer). ESI-MS (scan ES+, *m*/*z*): 795.14 (100%), 796.15 (40%), 797.15 (8%) ([Tb(bpcd)(Coum)] + Na)^+^.

### Luminescence and Decay Kinetics

Room-temperature luminescence was measured with a Fluorolog 3 (Horiba-Jobin Yvon) spectrofluorometer, equipped with a Xe lamp, a double-excitation monochromator, a single-emission monochromator (mod. HR320), and a photomultiplier in photon counting mode for the detection of the emitted signal. All of the spectra were corrected for the spectral distortions of the setup. The spectra in solution were recorded on methanol (50 μM) solutions.

In decay kinetics measurements, a xenon microsecond flash lamp was used and the signal was recorded by means of a multichannel scaling method. True decay times were obtained using the convolution of the instrumental response function with an exponential function and the least-squares-sum-based fitting program (SpectraSolve software package).

### Circularly Polarized Luminescence

CPL spectra were recorded with the homemade spectrofluoropolarimeter described previously.^31^ The spectra were recorded on methanol (0.4 mM) solutions in a 1 cm cell. The samples were excited at 365 nm {for [Eu(bpcd)(tta)]} or 254 nm {for [Eu(bpcd)(Coum)] and [Tb(bpcd)(Coum]}.

### Electronic Circular Dichroism (ECD)

ECD spectra were recorded with a Jasco J1500 spectropolarimeter on 1 mM CH_3_OH solutions in a 0.01 cm cell.

### Overall Quantum Yield Measurements

Overall quantum yields were measured by adopting the relative method. Fluorescein in 0.1 M NaOH (fluorescence quantum yield of 0.9) was used as the standard. Absorption spectra were recorded with a PerkinElmer Lambda 650 UV–vis spectrophotometer. Emission spectra were recorded with an Edinburgh FLS1000 fluorometer and corrected for excitation intensity and detector sensitivity. The samples were dissolved in methanol, while keeping their absorbance lower than 0.1. We obtained the same values of overall quantum yield for the two enantiomers.

### Spectrophotometric Titrations

The UV–vis absorption spectra were recorded in the wavelength range of 220–500 nm on a Varian Cary 50 spectrophotometer using 1 cm optical path length quartz cells (Hellma Analytics) sealed with a Teflon stopper. The samples were prepared in dry methanol within a drybox with a N_2_ atmosphere.

In our experiments, solutions of tta or Coum (initial concentrations of 5.0 × 10^–5^ mol L^–1^) were titrated with a [Eu(bpcd)]Cl solution (1.0 × 10^–3^ mol L^–1^) with a final ligand:complex molar ratio ∼3.0. The cell was stirred for ∼10 min before each spectrum was recorded. The formation constants of the [Eu(bpcd)(tta)] and [Eu(bpcd)(Coum)] complexes were obtained by multiwavelength analysis of the absorption spectra using HypSpec.^32^

### DFT Calculations

All molecular structures of the complexes were obtained by means of DFT calculations performed in Gaussian 16 (version A.03).^33^ In previous works,^30,34^ the paramagnetic Eu(III) ion was replaced by Y(III), which was a suitable substitute. This choice is also supported for the isostructural complexes found with analogous hexadentate ligands EDTA and CDTA. In the crystal structures with the latter two ligands,^35−37^ Y(III) and Eu(III) ions are nine-coordinated with EDTA (ligand and three water molecules bound to the metal) and eight-coordinated with CDTA (two bound water molecules). Because the ionic radius of Y(III) is smaller than those of Eu(III) and Tb(III), the calculations for the same complexes with the larger La(III) ion were also carried out. To identify the solvent molecules bound to the complexes, the geometries of [Ln(bpcd)(Coum)]·4H_2_O (L = Y or La), in which water molecules replaced methanol, were also considered.

The functional B3LYP^38,39^ was used with the 6-31+G(d) basis set for all ligand atoms and MWB28 pseudopotential and valence electron basis set for the metal ions.^40,41^ Geometry optimizations were carried out at the DFT level with a polarizable continuum model (PCM) to simulate solvation.^42^

As in a previous work,^43^ the excited state (T_1_ and S_1_) energies were obtained by employing the time-dependent DFT approach (TD-DFT) on the [Y(bpcd)L] complexes using the same level of theory as in the geometry optimizations, as it was shown that the B3LYP functional accurately predicts the UV–vis spectra of coumarin derivatives.^44^ Analyses were performed with Multiwfn version 3.8.^45^

## Results and Discussion

### Synthesis of the Complexes

Both enantiomers (*S,S* or *R,R*) of [Eu(bpcd)(tta)], [Eu(bpcd)(Coum)], and [Tb(bpcd)(Coum)] complexes have been obtained in very high chemical yields (∼95%) and high degrees of purity, as confirmed by the elemental analysis and ESI-MS data. Additional details of the synthesis are reported in the Experimental Section.

### UV–Vis Absorption and ECD

The UV–vis electronic absorption spectra and ECD spectra of the [Eu(bpcd)(tta)], [Eu(bpcd)(Coum)], and [Tb(bpcd)(Coum)] complexes in methanol are shown in Figure S1.

As it is well-known, the absorption band around 350 nm, in the case of [Eu(bpcd)(tta)], can be attributed to the diketonate-centered singlet–singlet π–π* transition of the tta ligand^46^ while the absorption band peaking around 270 nm is assigned to electronic transitions involving both pyridine ring (i.e., π–π* and n−π*) transitions of the bpcd^2–^ ligand.^30^ The lower-energy ECD bands indicate that the chiral ligand can dictate a preferred sense of twist of the diketonate, as demonstrated by a dichroic signal around 350 nm, where the absorption of tta takes place.

The UV–vis electronic absorption spectra of [Eu(bpcd)(Coum)] and [Tb(bpcd)(Coum)] complexes are practically identical, demonstrating the ligand-centered nature of the electronic transitions, which is independent of the type of metal ion. The absorption peaks centered around 230, 300, and 320 nm, as previously demonstrated,^27^ are related to Coum electronic transitions, while the shoulder at 270 nm is due to the absorption of the bpcd^2–^ ligand. Although both ECD spectra show an overall similar trend, the dichroic signals show some differences in intensity and shape especially in the range of 280–350 nm. Possibly, this could be due to the different size of the lanthanide cation (Eu^3+^ vs Tb^3+^), which in turn impacts the position of the Coum moiety relative to the bpcd ligand. Geometrical variations within a series of analogue lanthanide complexes are possible, especially across the so-called Gd break. For comparison, the ECD of the [Gd(bpcd)(Coum)] analogue was also measured. It displayed a trend similar to that shown by the Eu complex but with a somehow stronger band at 270 nm (Figure S1). Again, the CD signals in the range of 280–350 nm, which are associated with the achiral coumarin, indicate a stereochemically defined arrangement of the whole complex, including the coumarin.

### UV–Vis Titrations

**Figure 5:**
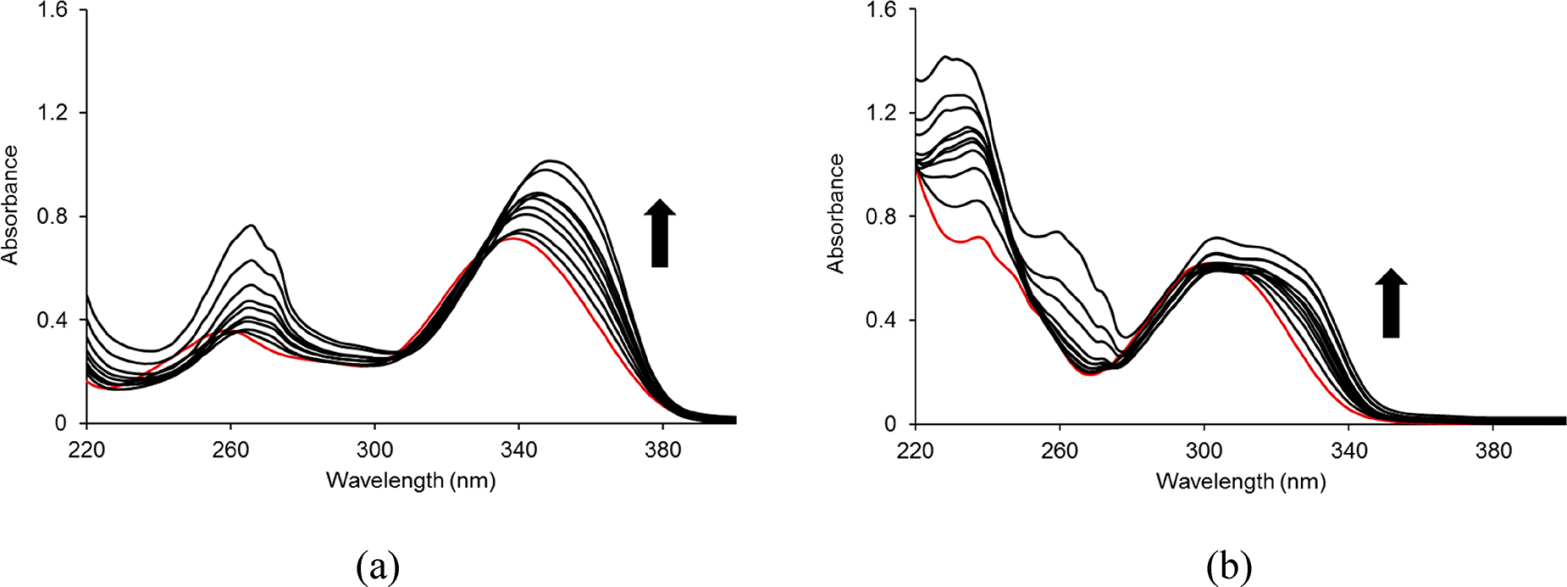
Changes in the UV–vis absorption spectra during the titration of (a) tta (50 μM) and (b) Coum (50 μM) upon addition of a solution of [Eu(bpcd)Cl] at 25 °C. The spectra of the initial tta and Coum solutions are colored red.

The absorption spectra of tta/Coum with an increasing concentration of [Eu(bpcd)]Cl were recorded in anhydrous methanol between 220 and 400 nm. The spectra of both systems present a maximum of absorption at 266 nm related to the pyridine group of the [Eu(bpcd)]^+^ complex.^30^ As for tta (Figure 5a), the initial absorption maximum at 340 nm undergoes a marked red-shift upon formation of the [Eu(bpcd)(tta)] complex in solution. On the contrary, for Coum (Figure 5b), the intensity of the initial absorption at 310 nm increases with a slight red-shift and a shoulder at 320 nm. The data were analyzed by simultaneous least-squares fitting of the absorbance data in the wavelength range associated with the formation of the new species (300–380 and 290–340 nm for tta and Coum, respectively). As an example of the goodness of fit, calculated and experimental absorbances at selected wavelengths are reported in Figure S2. The fitting procedure confirms the formation of quite stable 1:1 species (Table 1). Interestingly, we show that the complex formed with Coum is more stable (∼1.5 log units) than that with tta. In previous works, the formation of the [EuL_*j*_]^3–*j*^ species was studied in water for tta (log *K*_1_ = 4.65, log β_2_ = 9.67, and log β_3_ = 12.0 at 25 °C in water)^47^ and mixed solvents for Coum (50% water/dioxane; log *K*_1_ = 3.92 and log β_2_ = 6.89 at 35 °C).^48^ However, our results (Table 1) are not comparable with the literature data for the 1:1 species. The reaction reported in those works indeed occurs between the solvated Eu(III) ion and the ligands, with the stepwise formation constants for the 1:3 [log *K*_3_ = 2.33 for the reaction Eu(tta)_2_ + tta ⇋ Eu(tta)_3_]^47^ and 1:2 [log *K*_2_ = 2.97 for the reaction Eu(Coum) + Coum ⇋ Eu(Coum)_2_] ratios,^48^ clearly higher, due to the weaker solvation of the metal complex and the ligands in methanol than in water.^49^

**Table 1:** Equilibrium Constants (log *K*) for the Formation of the [Eu(bpcd)]L Complexes in Anhydrous Methanol at 25 °C (L = tta or Coum) (charges omitted)

	log *K*	
	L = tta	L = Coum
[Eu(bpcd)] + L ⇋ [Eu(bpcd)L]	4.12 ± 0.05	5.59 ± 0.06

### Total Luminescence (TL), CPL, and Luminescence Decay Kinetics

#### [Eu(bpcd)(tta)]

The luminescence excitation and emission spectra of a methanol solution (50 μM) of the complex are shown in Figure S3. The excitation spectrum is very similar to the absorption spectrum, and the presence of two excitation peaks at 270 and 350 nm is related to an efficient ligand-to-metal energy transfer involving both ligands. The emission spectrum is dominated by the presence of the hypersensitive ^5^D_0_ → ^7^F_2_ transition of Eu(III), which is usually observed when the emitting Eu(III) is located in a site, whose point symmetry lacks the inversion center. The high value (9.54) of the asymmetry ratio
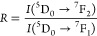
1is compatible with a highly distorted geometric environment around the metal ion.

**Figure 6:**
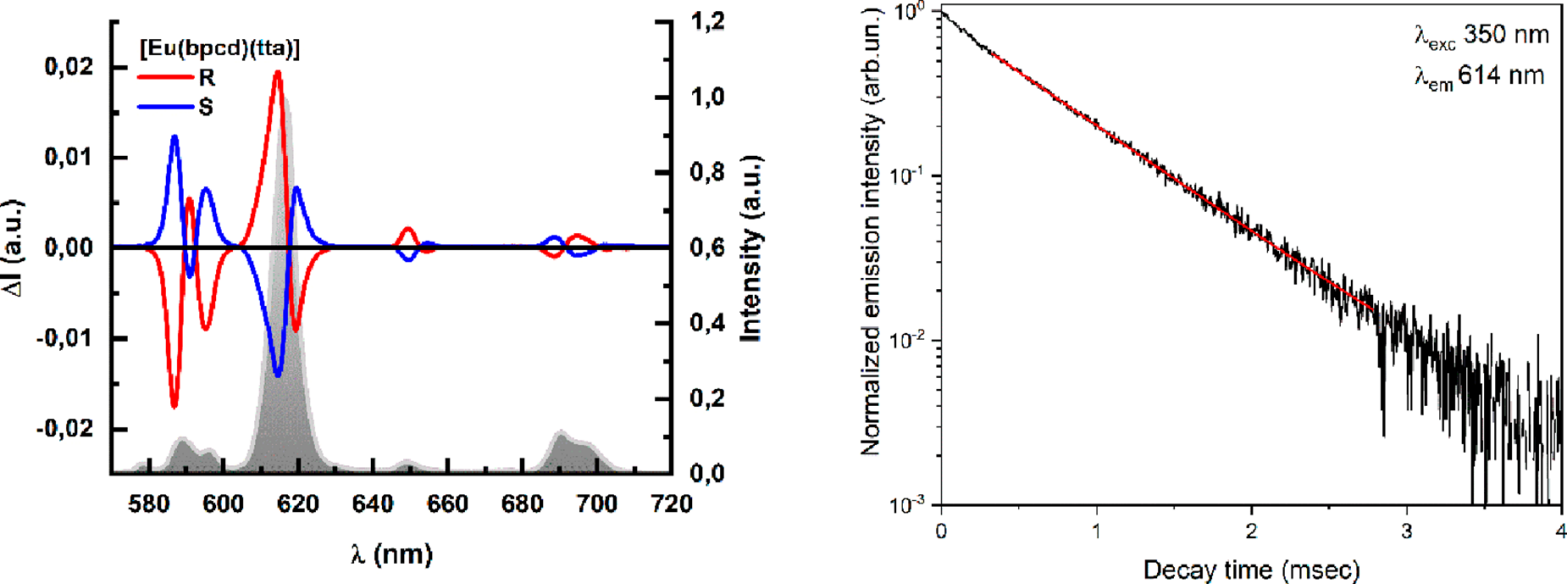
CPL spectra (left) of both enantiomers of [Eu(bpcd)(tta)], with the normalized total luminescence spectrum overlaid, upon excitation at 365 nm. Eu(III) ^5^D_0_ luminescence decay curve (right) (the plot of the *S,S* isomer is shown and chosen as a representative). The equation of the fitting curve (red line) is *y* = 0.903 exp(−*t*/0.68) + 0.001 [reduced χ^2^ = 4.0 × 10^–5^; *R*^2^ (COD) = 0.99803].

The luminescence decay curve of the ^5^D_0_ excited state of Eu(III) in methanol can be properly fitted by a single-exponential function (Figure 6, right). The calculated observed lifetime [0.68(1) ms] is reported in Table 2.

**Table 2:** Most Relevant Photophysical Parameters of the Eu(III) and Tb(III) Complexes in Methanol Investigated in This Work^a^

complex	τ_obs_ (ms)	τ_obs_ (ms), CD_3_OD	τ_rad_ (ms)	ϕ_int_ (%)	ϕ_ovl_ (%)	η_sens_ (%)	*m*^b^
[Eu(bpcd)(tta)]	0.68(1)	1.18(1)	2.38(1)	26.4(1)	26	100	1.3(5)^c^
[Eu(bpcd)(Coum)]	0.80(1)	1.40(1)	2.42(1)	33.1(1)	7	21.1	1.1(5)^c^
[Tb(bpcd)(Coum)]	1.42(1)	1.59(1)	–	–	55	≥55^a^	0.6(5)^d^

a The reported values are the same for both enantiomers.

b Number of methanol molecules in the inner coordination sphere.

c Calculated as described in ref (50). Following the Judd–Ofelt theory,^56^ one can calculate τ_rad_ from the emission spectra, only in the case of the Eu(III) ion.

d Calculated by a modification for Tb(III) of the equation discussed for the Eu(III)^50^ ion.

To estimate the number of methanol molecules in the inner coordination sphere of the Eu(III) ion (*m*), the observed lifetime was also measured in CD_3_OD (1.18 ms). The value obtained here for *m* is 1.3(5) and has been determined by using the equation *m* = 2.1(1/τ_MeOH_ – 1/τ_CD_3_OD_) (Table 2).^50^ This number has a significant impact on the luminescence efficiency of the emitting lanthanide ion. In fact, the OH group of methanol possesses high-energy vibrations (3300–3400 cm^–1^), which are particularly effective in the nonradiative quenching of the lanthanide emitting level, by means of the multiphonon relaxation process (MPR).^51^ A higher *m* value would lead to stronger quenching of the Ln(III) luminescence. By using the equation reported by Werts,^52^ which is known to be applicable to only the emission spectra of Eu(III), we calculated the radiative lifetime (τ_rad_). We also determined the intrinsic quantum yield (ϕ_int_ = τ_obs_/τ_rad_; i.e., the ratio of emitting/absorbed photons upon direct excitation into a luminescent level of the lanthanide ion) and the overall quantum yield upon excitation of the tta ligand (ϕ_ovl_; i.e., the ratio of emitting/absorbed photons upon excitation of the ligand) by using the secondary methods described in the literature^53^ and *η*_sens_, which is the overall energy transfer efficiency (*η*_sens_ = ϕ_ovl_/ϕ_int_). It is worth noting that the obtained value of *η*_sens_ is ∼100%, which underlines the high efficiency of the *antenna* effect of tta molecules in sensitizing the Eu(III) luminescence in the [Eu(bpcd)(tta)] complex.

The CPL spectra of [Eu(bpcd)(tta)] (Figure 6) show intense bands with *g* factors on the order of 10^–1^ and 10^–2^ for the ^5^D_0_ → ^7^F_1_ and ^7^F_2_ transitions, respectively (see Table 3 and Figure S4). Notably, three and two bands are clearly resolved for the ^5^D_0_ → ^7^F_1_ and ^7^F_2_ transitions, respectively, corresponding to the M_J_ splitting due to the crystal field.

**Table 3:** Photophysical Parameters and *B*_CPL_ Values of CPL-Active Eu(III) and Tb(III) Complexes Investigated in This Work

complex	ε (M^–1^ cm^–1^) [λ_abs_ (nm)]	ϕ_ovl_ (%)	|*g*_lum_| [λ (nm)]	*B*_CPL_ (M^–1^ cm^–1^)^a^
[Eu(bpcd)(tta)]	17000 (350)	26	0.26 (586)	13.2
			0.13 (595)	7.8
			0.02 (615)	22.3
[Eu(bpcd)(Coum)]	12000 (318)	7	0.06 (596)	1.4
			0.01 (614)	2.0
			0.01 (624)	0.8
[Tb(bpcd)(Coum)]	12000 (313)	55	0.05 (537)	11.7
			0.02 (547)	12.0
			0.04 (555)	6.9

a Calculated according to a modified formula (see the Supporting Information for more details).

#### [Eu(bpcd)(Coum)] and [Tb(bpcd)(Coum)]

The luminescence excitation and emission spectra of a methanol solution (50 μM) of the complexes are shown in Figure S3. Both excitation spectra are very similar to the corresponding absorption spectra. The presence of the excitation bands at 270 and 320 nm indicates, also for these Coum-based complexes, the presence of an efficient ligand-to-metal energy transfer involving both ligands. As in the case of the [Eu(bpcd)(tta)] complex, the emission spectrum of [Eu(bpcd)(Coum)] is dominated by the presence of the hypersensitive ^5^D_0_ → ^7^F_2_ transition of Eu(III) (Figure S3), although the geometric environment of the metal ion is less distorted than in the case of the tta-based complex, as suggested by the lower value of the asymmetry ratio (7.13). In the case of the [Tb(bpcd)(Coum)] complex upon excitation in the ligand absorption bands, the typical Tb(III) emission stemming from the *f*–*f* transition is detected (Figure S3).

**Figure 7:**
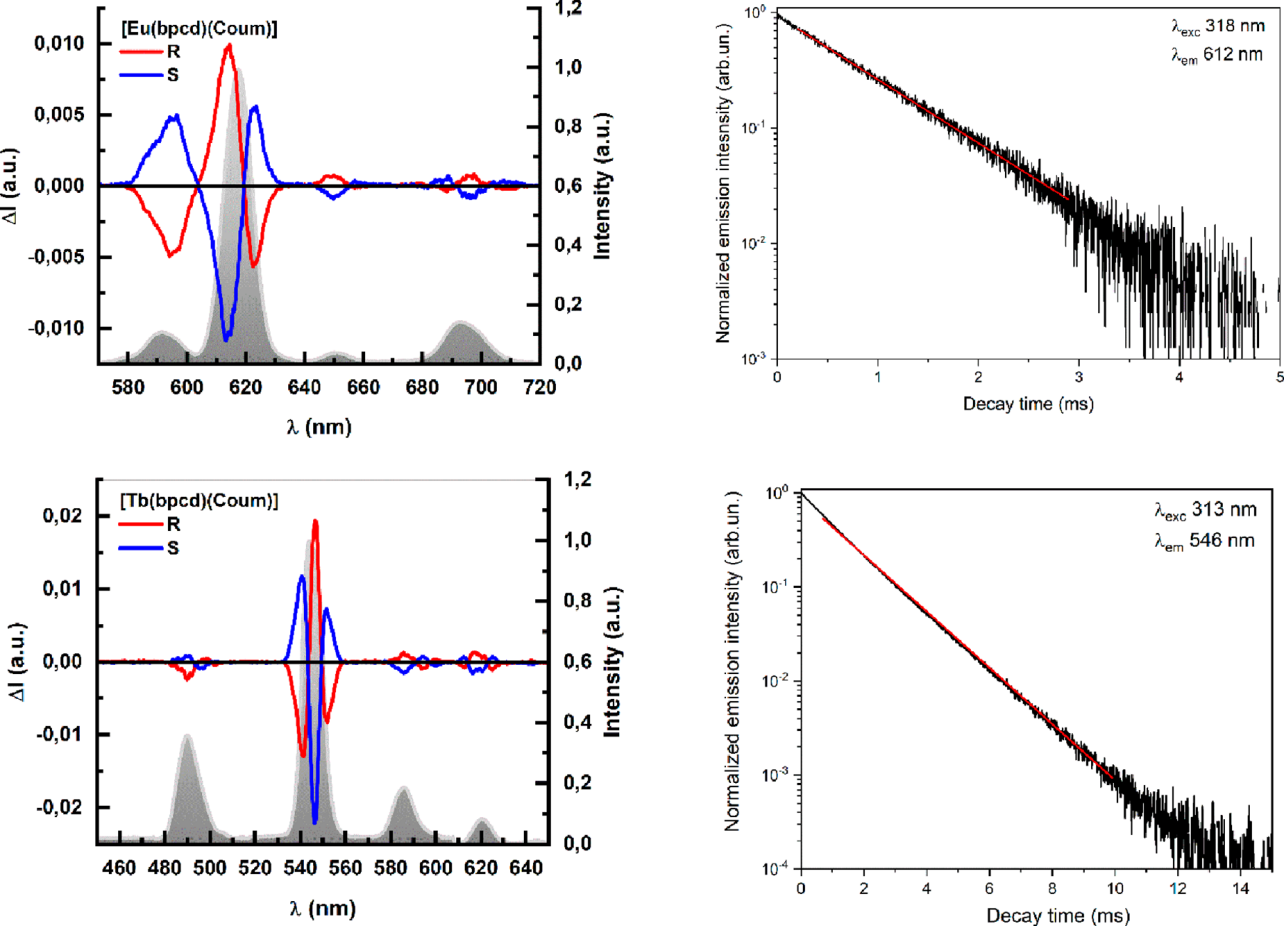
Overlap of the CPL and normalized total luminescence spectra (left) and luminescence decay curves (right) of [Eu(bpcd)(Coum)] (top) and [Tb(bpcd)(Coum)] (bottom) complexes. The luminescence spectra were recorded upon excitation at 254 nm. The decay curves of the *S,S* isomers are shown and chosen as a representative. The equations of the fitting curve (red line) are *y* = 0.93577 exp(−*t*/0.80) + 0.0004 [reduced χ^2^ = 1.6 × 10^–4^; *R*^2^ (COD) = 0.99531] for [Eu(bpcd)(Coum)] and *y* = 0.965 exp(−*t*/1.42) + 0.004 [reduced χ^2^ = 3.2 × 10^–6^; *R*^2^ (COD) = 0.99981] for [Tb(bpcd)(Coum)].

The luminescence decay curves of the excited states of Eu(III) and Tb(III) (^5^D_0_ and ^5^D_4_ levels, respectively) are fitted by a single-exponential function (Figure 7), and the calculated observed lifetimes (reported in Table 2) are 0.80(1) and 1.42(1) ms for the Eu(III) and Tb(III) complexes, respectively. As discussed above, the number of methanol molecules bound to the metal ion (*m*) can be obtained by measuring the observed lifetimes also in deuterated methanol. This number is slightly greater than 1 in the case of the [Eu(bpcd)(Coum)] complex and slightly less than 1 for [Tb(bpcd)(Coum)] (Table 2). A possible explanation for this behavior is given in DFT Calculations. A similar trend was observed by Arauzo et al.^54^ in heteroleptic Eu and Tb complexes containing the Coum and phenanthroline ligands. They observed the presence of one solvent molecule (H_2_O) in the inner coordination sphere for only the Eu(III) derivative, while the Tb(III) complex lacks a coordinated solvent. Interestingly, we can also conclude that the Coum ligand is not a good sensitizer for Eu(III) luminescence (as *η*_sens_ is only ∼21%) but can better sensitize Tb(III) luminescence [ϕ_ovl_ = 55%; η_sens_ ≥ 55 (Table 2)]. This statement is in agreement with previous works on Tb(III) and Eu(III) tris chelates of Coum,^27^ where the values of ϕ_ovl_ are 29% and 12%, respectively, and coumarin dipicolinate Eu(III) complexes (ϕ_ovl_ < 2%).^55^ When heteroleptic complexes of the Coum ligand contain phenanthroline or bathophenathroline ligands, the overall quantum yields increase for both Eu(III) (40–45%) and Tb(III) (58–76%).^54^ This feature is related to the involvement of the phenanthroline-based ligands in the sensitization of Ln(III) luminescence.

As expected, both CPL spectra of [Eu(bpcd)(Coum)] and [Tb(bpcd)(Coum)] (Figure 7) show mirror image spectra. Unlike the pattern of bands shown by [Eu(bpcd)(tta)] for the ^5^D_0_ → ^7^F_1_ transition, the coumarin complex has a single monosignate band (Figure 7). [Tb(bpcd)(Coum)] shows transitions associated with ^5^D_4_ → ^7^F_6,5,4,3_, with the most prominent feature being the manifold ^5^D_4_ → ^7^F_5_ transition around 547 nm. Despite the relatively low *g*_lum_ factors (Figure S6), because of its high quantum yield, [Tb(bpcd)(Coum)] shows high values of *B*_CPL_, comparable to those obtained for [Eu(bpcd)(tta)] (Table 3).

A survey of the literature data reveals that the values of ϕ_ovl_ and *g*_lum_ of [Eu(bpcd)(tta)] and of *B*_CPL_ for both [Tb(bpcd)(Coum)] and [Eu(bpcd)(tta)] complexes are in line with the average values reported for Eu(III) and Tb(III) luminescent complexes.^57^

### DFT Calculations

**Figure 8:**
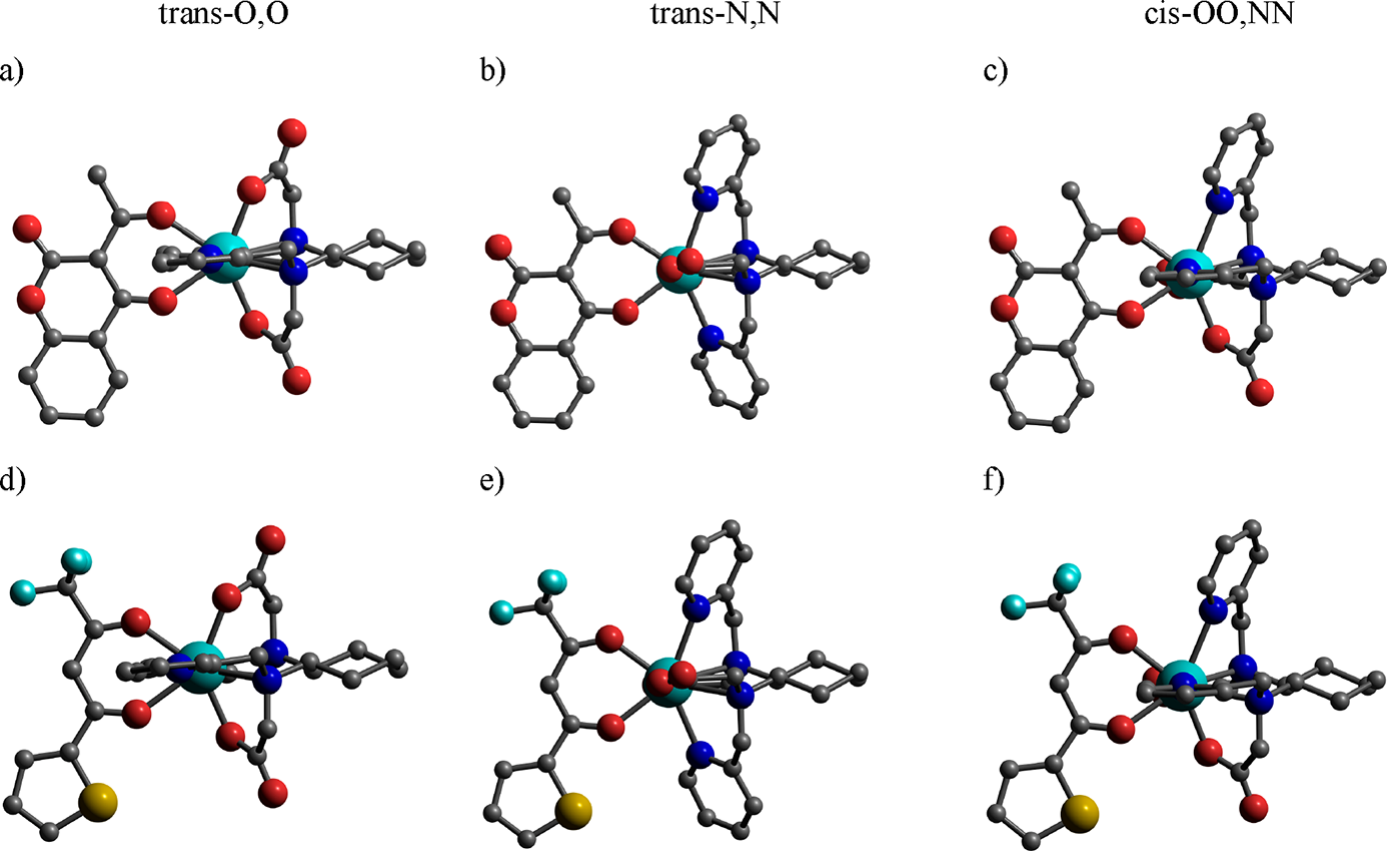
Minimum energy structures of the (a–c) [Y(bpcd)(Coum)] and (d–f) [Y(bpcd)(tta)] complexes obtained from DFT calculations. Hydrogen atoms bound to carbons have been omitted for the sake of clarity.

DFT calculations were carried out to obtain structural information about the [Ln(bpcd)L] complexes by studying the diamagnetic analogues of the Eu and Tb complexes. As previously described,^30,58^ several isomeric forms can be present, depending on the arrangement of the pyridine and acetate groups in the bpcd ligand, namely, *trans*-OO, *trans*-NN, and *cis*-OO,NN. These isomers can host either the Coum (Figure 8a–c) or tta (Figure 8d–f) ligand, which replaces the coordinated solvent molecules. The energy of the isomers does not differ significantly (Δ*E* < 0.3 and 1.0 kcal mol^–1^ for the adduct with Coum and tta, respectively), so that a mixture should be present in solution.

In our previous work,^30^ the number of water molecules coordinated to the metal ion in the structures of [Y(bpcd)H_2_O_*n*_]^+^ (*n* = 2–5) complexes was always 2. The additional waters remained dissociated and formed hydrogen bonds with the ligand or the coordinated ones. Nevertheless, a similar calculation for the [La(bpcd)H_2_O_*n*_]^+^ counterpart showed that more than two water molecules could interact directly with the metal ion.

**Figure 9:**
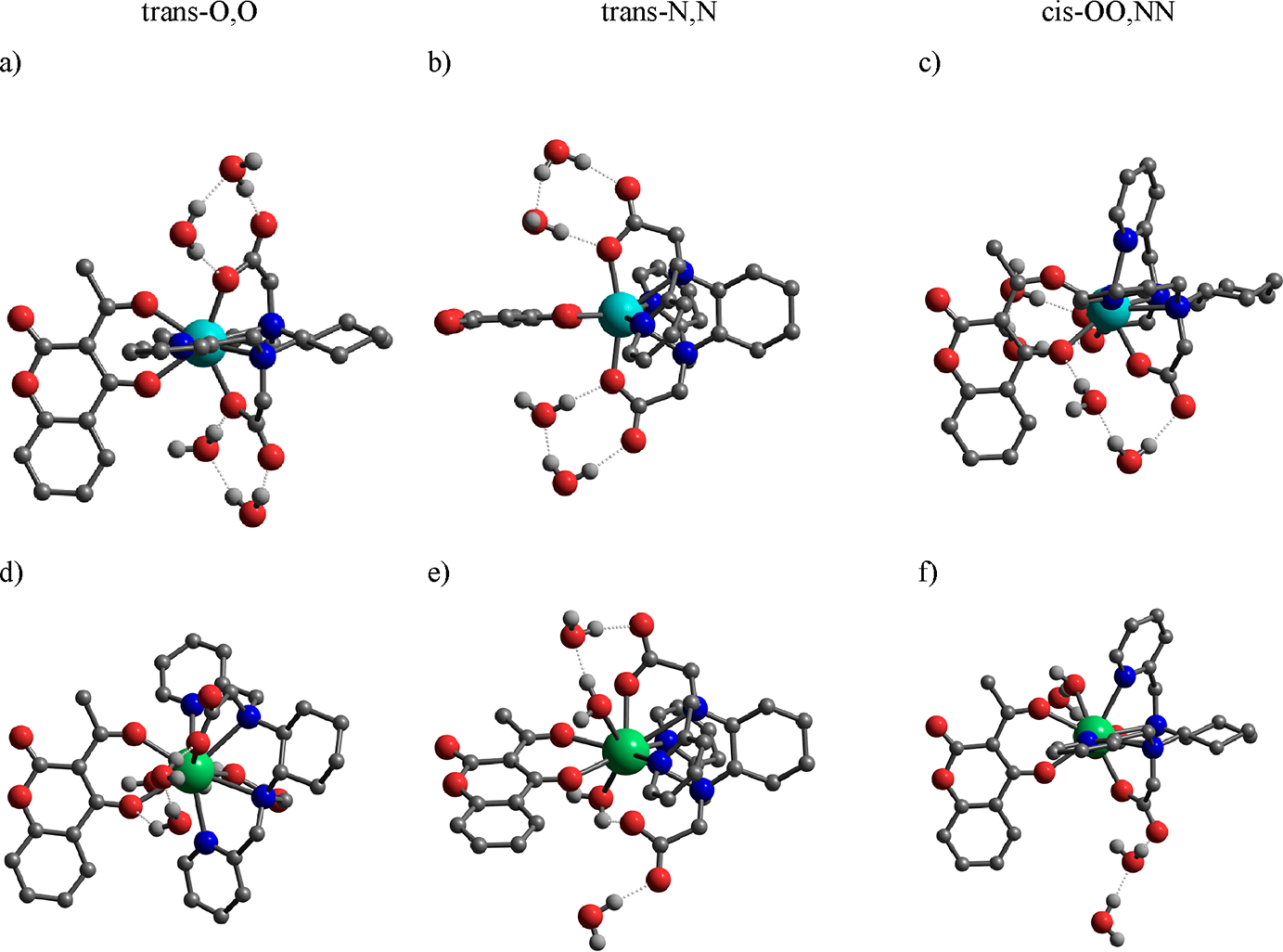
Minimum energy structures of the (a–c) [Y(bpcd)(Coum)]·4H_2_O and (d–f) [La(bpcd)(Coum)]·4H_2_O complexes obtained from DFT calculations. Hydrogen atoms bound to carbons have been omitted for the sake of clarity.

The calculated number of coordinated methanol molecules in this study is 1.3 and 1.1 for Eu(III) and 0.6 for Tb(III). To confirm the solvent coordination, we considered the [Y(bpcd)(Coum)]·4H_2_O complexes, in which water was employed in place of methanol to reduce the number of degrees of freedom of the system. The first result is that water cannot interact with the metal in all [Y(bpcd)(Coum)]·4H_2_O isomers (Figure 9a–c). Second, the differences in energy [Δ*E* = *E*(isomer) – *E*(a)] are 1.2 and 4.6 kcal mol^–1^ for structures b and c, respectively, in agreement with our previous observation that the *cis*-OO,NN isomer was the less stable one.

In the obtained minimum energy structures, the shortest metal–O_water_ distances fall in the range of 4.2–4.5 Å. Even if it has been proposed that closely diffusing solvent molecules (like those shown in Figure 9a–c) can contribute to decrease the luminescence decay lifetime, the number of methanol molecules calculated from the experimental measurements is definitely greater than 1 for Eu(III).

In the [La(bpcd)(Coum)]·4H_2_O structures (Figure 9d–f), on the contrary, two water molecules can coordinate to the metal ion in the *trans*-OO and *trans*-NN isomers (La–O_water_ bond lengths of 2.64–2.97 Å), while one is found in the *cis*-OO,NN form (La–O_water_ distance of 2.77 Å). The energy difference between the different structures is <2.8 kcal mol^–1^, which suggests the presence of a mixture in solution. Because La(III) and Y(III) have ionic radii, longer and shorter than that of Eu(III),^b^ respectively, it is likely that the small differences allow the coordination of an additional solvent molecule in solution. Interestingly, the experimental *m* (Table 2) clearly decreases from Eu (1.3 or 1.1) to Tb (0.6), in agreement with the hypothesis that small changes in ionic radius here determine a different number of coordinated solvent molecules in the complex. Therefore, in this case, Y(III) models are more representative of the [Tb(bpcd)(Coum)] coordination in solution.

The energies of the S_1_ and T_1_ excited states for both tta and Coum ligands were determined by TD-DFT calculations for the Y- and La-based complexes (Table 4). The HOMO and LUMO molecular orbitals, mainly localized on these two ligands, are mostly involved in the S_0_ → S_1_ and S_0_ → T_1_ transitions (Figures S7–S10), and the two most intense peaks at lower energies [around 315 nm for Coum-based complexes and around 350 nm for tta-based complexes (Figures 5 and 6)] are related to these S_0_ → S_1_ transitions. As previously demonstrated by some of us, at shorter wavelengths (at 270 nm), an additional electronic transition involving the molecular orbitals localized on the bpcd ligand can be exploited to sensitize Eu(III)^43^ and Tb(III)^30^ luminescence (not discussed here).

**Table 4:** Energies of the S_1_ and T_1_ States (cm^–1^) Obtained from TD-DFT Calculations and Estimated Donor–Acceptor Distances, *R*_L_ (Å), Corresponding to the Unoccupied Molecular Orbitals Centroid to the Metal Ion

complex	S_1_	T_1_	difference	*R*_L_
[Y(bpcd)(Coum)]				
*trans*-O,O	31514	25636	5878	3.37
*trans*-N,N	31865	25606	6259	3.47
*cis*-O,O-N,N	31769	25612	6157	3.28
[Y(bpcd)(tta)]				
*trans*-O,O	29008	19640	9368	3.51
*trans*-N,N	29138	19582	9556	3.54
*cis*-O,O-N,N	29122	19631	9491	3.50
[La(bpcd)(Coum)(H_2_O)_*n*_]				
*trans*-O,O (*n* = 1)	32614	26033	6581	3.98
*trans*-N,N (*n* = 2)	32552	25882	6670	4.08
*cis*-O,O-N,N (*n* = 1)	32045	25632	6413	3.83
[La(bpcd)(tta)(H_2_O)_*n*_]				
*trans*-O,O (*n* = 1)	29674	20007	9667	3.89
*trans*-N,N (*n* = 2)	29671	20026	9645	3.96
*cis*-O,O-N,N (*n* = 1)	29179	19512	9667	3.76

Assuming the participation of the excited triplet state in the *antenna* process, the sensitization of Tb(III) luminescence by tta can be ruled out. In fact, the lowest excited state of Tb(III) (^5^D_4_) is located around 20500 cm^–1^; that being above the energy of the triplet state of the ligand (19618 cm^–1^ averaged on the possible isomers) could be responsible for a back energy transfer mechanism (from the metal to the ligand) that makes the *antenna* process ineffective. On the contrary, the energy position of the T_1_ state of tta seems to be optimal to transfer energy to the ^5^D_0_ emitting level of Eu(III) (located at 17300 cm^–1^), in agreement with the calculated η_sens_ of ∼100% (Table 2). As for the energy transfer mechanism in the case of the Coum ligand, both the emitting ^5^D_4_ level of Tb(III) located at 20500 cm^–1^^59^ and the ^5^D_2_ level of Eu(III) (around 21500 cm^–1^) can be considered suitable acceptor states of the T_1_ donor state of the Coum molecule [around 25600/26000 cm^–1^ (Table 4)]. However, in our complexes, the sensitization efficiency of Eu(III) luminescence by the Coum ligand is lower than that of Tb(III) {η_sens_ values around 20% and ≥55% for the [Eu(bpcd)(Coum)] and [Tb(bpcd)(Coum)] complexes, respectively}. Although a detailed study of the dynamics of the energy transfer process would be necessary to clearly understand the reasons underlying this behavior, a possible explanation for the lower sensitization efficiency in the case of the Eu(III) complex can be found in the D–A (donor–acceptor) distance (*R*_L_) and the S_1_–T_1_ energy gap (fifth and fourth columns, respectively, in Table 4). In fact, it is well-known that the *R*_L_ distance strongly affects the probability of energy transfer from D to A: the shorter the *R*_L_, the higher the probability.^60^ As it can be evinced from the inspection of Table 4, with an increase in the ionic radius of the lanthanide ion (passing from Y to La), the D–A distance increases. Therefore, we expect a sensitization efficiency that improves as the lanthanide ion becomes smaller. The decrease (|Δ*r*| = 0.03 Å) in the ionic radius (and in turn of the *R*_L_ distance) by passing from the Eu(III) to Tb(III) ion can justify the higher sensitization efficiency observed in the case of the Tb(III)-based complex. Analogously, it seems that an increase in the S_1_–T_1_ energy gap occurs with an increase in the size of the metal ion [from Y to La (Table 4) and presumably from Tb to Eu]. The increase in this energy gap is often related to a decrease of the intersystem crossing and sensitization efficiencies. Finally, as usual for lanthanide-based coordination compounds, in the energy transfer process we can assume the exchange mechanism as dominant, being the most sensitive to the D–A distance.^60^ Therefore, its involvement could account for the significant decrease in sensitization efficiency, even if only a very small increase in the ionic radius (and in turn the *R*_L_ distance) occurs by passing from the Tb(III) to Eu(III) ion.

## Conclusions

Upon excitation of the *antenna* ligands, a moderate to good overall quantum yield is measured for the [Eu(bpcd)(tta)] (26%) and [Tb(bpcd)(Coum)] (55%) complexes. This is mainly due to the very good sensitization efficiency of the Eu(III) and Tb(III) luminescence by tta and Coum *antenna* ligands, respectively. A shorter D–A distance and a better intersystem crossing efficiency, in the case of [Tb(bpcd)(Coum)], could account for the observed higher sensitization efficiency of the Coum ligand toward Tb(III) (≥55%), with respect to Eu(III) (21%). In addition, the access to the first coordination sphere of the metal ion by the methanol molecules, which decrease the luminescence efficiency, is more limited in the case of the Tb(III) complex (*m* = 0.6) than in the case of the Eu(III) complex (*m* = 1.1). This is probably due to an ionic size effect, Eu(III) being slightly larger than Tb(III) (Δ*r* = 0.03 Å; *r* is the ionic radius in water). As suggested by the D–A distances (*R*_L_), the Coum ligand is more tightly bound to the metal ion than tta, and this reflects the fact that the stability of [Eu(bpcd)(Coum)] (log *Κ* = 5.59) is higher than that of [Eu(bpcd)(tta)] (log *Κ* = 4.12). The ECD experiments suggested that the chiral bpcd ligand can dictate a preferred sense of twist of both tta and Coum ligands and the CPL activity of the magnetic dipole transition around 586 nm of Eu(III) in [Eu(bpcd)(tta)] complex is remarkable (*g*_lum_ = 0.26). The reported values of *B*_CPL_ for these complexes are in line with the average values reported in the literature for Eu(III) and Tb(III) luminescent complexes.

## Supporting Information Available

Excitation and emission spectra and plots of *g*_lum_ versus wavelength of the Eu(III) and Tb(III) complexes under investigation; Kohn–Sham molecular orbital composition of the S_1_ and T_1_ states for the Y(III) and La(III) counterparts; additional experimental details, materials, and methods of the chiroptical instrumentation and definition of the *B*_CPL_ formula employed in this work; and ^1^H and ^13^C NMR data of the Coum ligand (PDF)

## Footnotes

a Because (i) ϕ_int_, ϕ_ovl_, and η_sens_ can assume values between 0 and 1, (ii) ϕ_ovl_ must be less than or equal to ϕ_int_, and (iii) ϕ_ovl_ is 55% for the [Tb(bpcd)(Coum)] complex, we can conclude that η_sens_ must be ≥55%.

b The recently revised^61^ ionic radii in a water solution are 1.250 (1.216), 1.12 (1.066), and 1.090 (1.04) Å for La(III), Eu(III), and Tb(III), respectively (Shannon radii^62^ in parentheses). For Y(III), the ionic radius calculated from the Y–O(water) distance obtained experimentally^63^ using the water radius proposed in ref (61) is 1.020 (1.019) Å.
