# Supplementary material for: Circularly Polarized Luminescence from New Heteroleptic Eu(III) and Tb(III) Complexes
Source: Inorg Chem. 2023 Jun 1;62(23):8812–22. doi: 10.1021/acs.inorgchem.3c00196 (PMC10265700; doi:10.1021/acs.inorgchem.3c00196)
Supplement: Supplementary file 1 — ic3c00196_si_001.pdf [file ic3c00196_si_001.pdf]

Supporting information

Circularly Polarized Luminescence from new heteroleptic Eu(III) and Tb(III) complexes

Silvia Ruggieri,[1] Silvia Mizzoni,[1] Chiara Nardon,[1] Enrico Cavalli,[2] Cristina Sissa,[2] Michele Anselmi,[3] Pier Giorgio Cozzi,[3,4] Andrea Gualandi,[3,4] Martina Sanadar,[5] Andrea Melchior,[5*] Francesco Zinna,[6] Oliver G. Willis,[6] Lorenzo Di Bari,[6*] Fabio Piccinelli[1*]

[1] Luminescent Materials Laboratory, DB, University of Verona, and INSTM, UdR Verona, Strada Le Grazie 15, 37134 Verona, Italy

[2] Department of Chemistry, Life Sciences and Environmental Sustainability, Parma University Parco Area delle Scienze, 17/a – 43124, Parma, Italy

[3] Department of Chemistry "G. Ciamician", University of Bologna, via Selmi 2 – 40126, Bologna, Italy

[4] Center for Chemical Catalysis - C3, Alma Mater Studiorum - Università di Bologna, Via Selmi 2, 40126 Bologna, Italy

[5] Dipartimento Politecnico di Ingegneria e Architettura, Laboratorio di Tecnologie Chimiche, Università di Udine, via Cotonificio 108, 33100 Udine, Italy

[6] Department of Chemistry and Industrial Chemistry, University of Pisa, via Moruzzi 13, 56124 Pisa, Italy

*corresponding authors' email address: fabio.piccinelli@univr.it, lorenzo.dibari@unipi.it and andrea.melchior@uniud.it

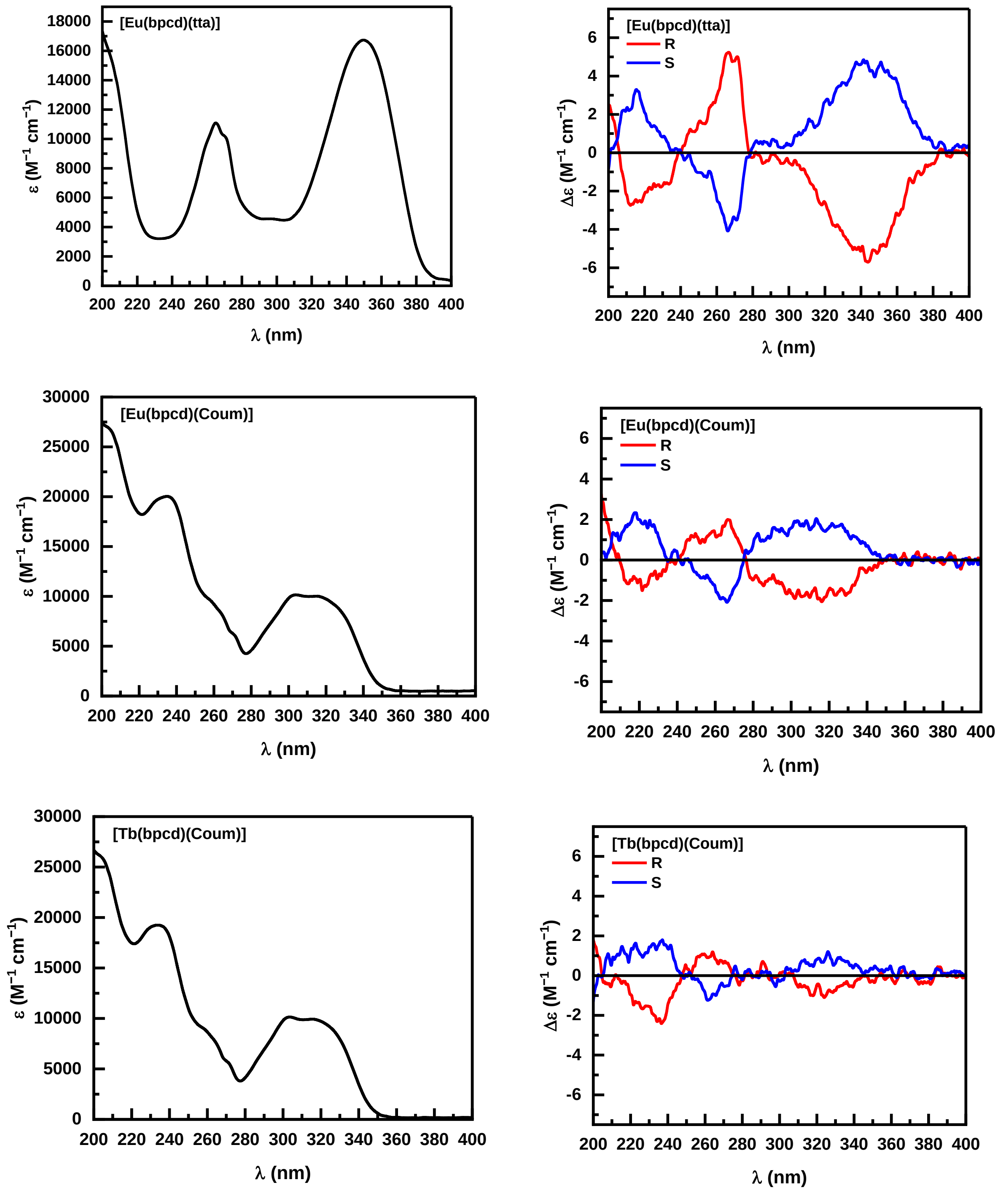
[Eu(bpcd)(tta)]
ε (M^{-1} cm^{-1})
λ (nm)
[Eu(bpcd)(tta)]
R
S
Δε (M^{-1} cm^{-1})
λ (nm)
[Eu(bpcd)(Coum)]
ε (M^{-1} cm^{-1})
λ (nm)
[Eu(bpcd)(Coum)]
R
S
Δε (M^{-1} cm^{-1})
λ (nm)
[Tb(bpcd)(Coum)]
ε (M^{-1} cm^{-1})
λ (nm)
[Tb(bpcd)(Coum)]
R
S
Δε (M^{-1} cm^{-1})
λ (nm)

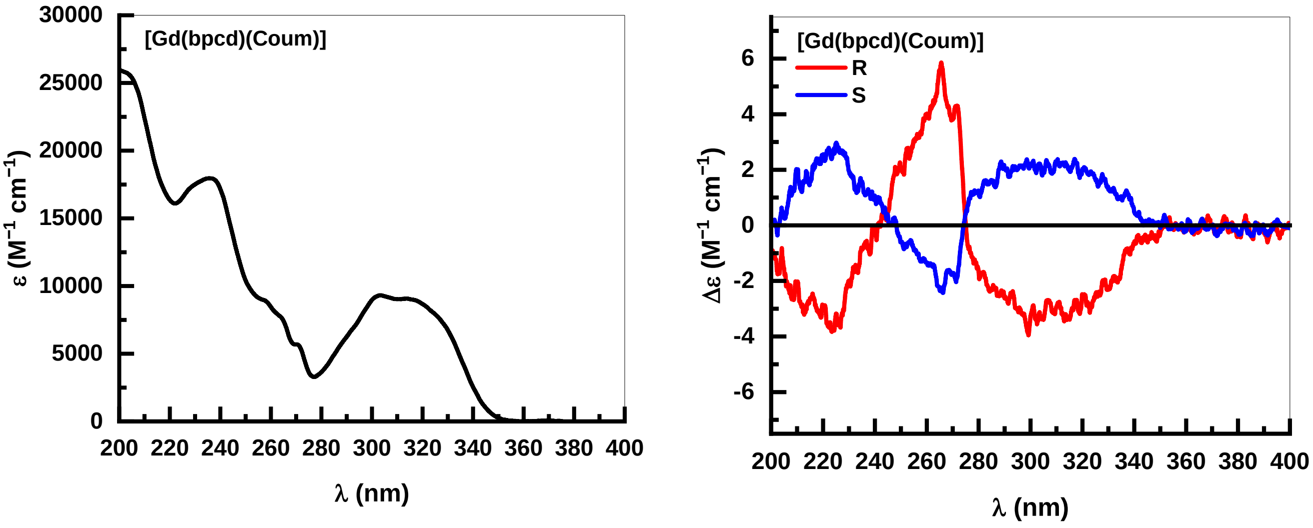

Figure S1. Absorbance of both the enantiomers reported as the average of their molar extinction coefficients (ε) (left) and ECD (right) spectra of [Eu(bpcd)](tta) (row 1), [Eu(bpcd)(Coum)] (row 2), [Tb(bpcd)(Coum)] (row 3) and [Gd(bpcd)(Coum)] (row 4). The spectrum of the *R,R* enantiomer is reported in red while the spectrum of the *S,S* enantiomer is reported in blue.. The concentration of all the enantiomers is 1 mM and the spectra were measured with 0.01 cm path length.

a)

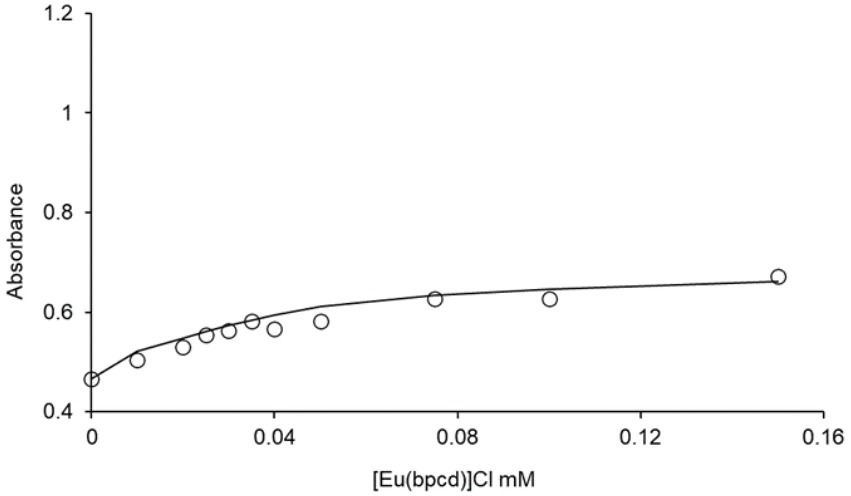

b)

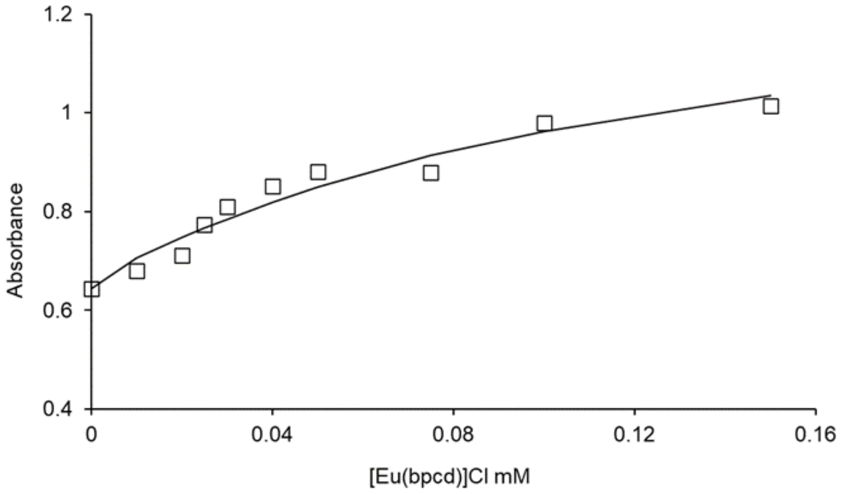

Figure S2. Experimental absorbance data (symbols) and fitting isotherm obtained by least squares-treatment in Hypspec for the spectra recorded upon addition of [Eu(bpcd)]Cl to 50 μM solutions of a) Coum (λ= 317 nm) and b) tta (λ = 347 nm).

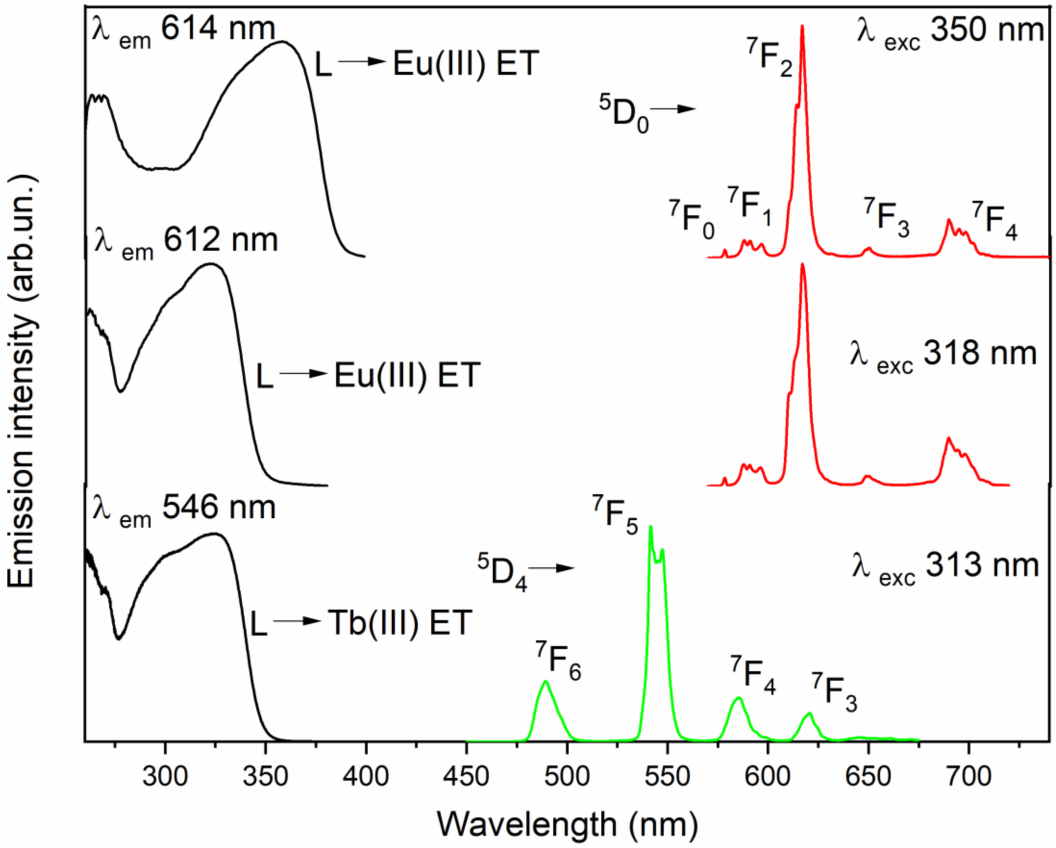

Figure S3. Luminescence excitation (left) and emission (right) spectra of (*S*,*S*)-[Eu(bpcd)(tta)] (top); (*S*,*S*)-[Eu(bpcd)(Coum)] (middle) and (*S*,*S*)-[Tb(bpcd)(Coum)] (bottom) complexes dissolved in methanol (50 μM). The spectra of the *R*,*R* enantiomer are not reported as they are perfectly comparable to those of the *S*,*S* counterpart.

Chiroptical instrumentation

CPL measurements

Discrimination of left/right circular polarized states was performed by a photoelastic modulator from a decommissioned Jasco J500C spectropolarimeter operating at 50 KHz coupled with an uncoated Glenn-Thompson polarizer. A Jasco CT-10 was used as the emission monochromator and the detection was performed by a Hamamatsu R376 PMT. The spectra were collected under either 365 or 254 nm irradiation from commercial LED-sources, using a 90° geometry between the excitation and detection direction. All CPL spectra were recorded in 1 cm semi-micro (aperture 4 mm) optical glass cells using the following parameters: scan-speed 0.5 nm/sec, integration time 2 sec, photomultiplier tube driving voltage 600 V, accumulations 4.

UV-Vis/ECD measurements

UV-Vis spectra were recorded using a Jasco-V650 spectrophotometer in the spectral range of 200 to 400 nm. All samples were measured in 1 mM MeOH solutions at room temperature. The same solutions were used to record CD spectra using a J1500 spectropolarimeter in 0.01 cm optical glass cells.

B_{CPL}

Given the complex structure of CPL spectra with several opposite bands within the same manifold, a more general definition of B_{CPL} factor was employed:

$$B_{CPL} = \varepsilon \cdot \varphi \cdot \frac{1}{2} \frac{\int_{\lambda_a}^{\lambda_b} I(\lambda) g(\lambda) d\lambda}{\int_{\lambda_i}^{\lambda_f} I(\lambda) d\lambda}$$

Where the integral in the numerator has to be estimated between the extrema of the considered transition (λ_a, λ_b), while the integral in the denominator is calculated over the whole emission range (λ_i, λ_f). Note that if g(λ) does not change sign within a term-to-term transition and it is approximately constant ($g(\lambda) \simeq \bar{g}$), then the above definition is reduced to:

$$B_{CPL} = \varepsilon \cdot \varphi \cdot \frac{1}{2}\bar{g} \cdot \frac{\int_{\lambda_a}^{\lambda_b} I(\lambda)d\lambda}{\int_{\lambda_i}^{\lambda_f} I(\lambda)d\lambda} = \varepsilon \cdot \varphi \cdot \frac{1}{2}\bar{g} \cdot \beta$$

According to the usual definition applicable to lanthanide CPL.[S1]

Additional chiroptical spectra

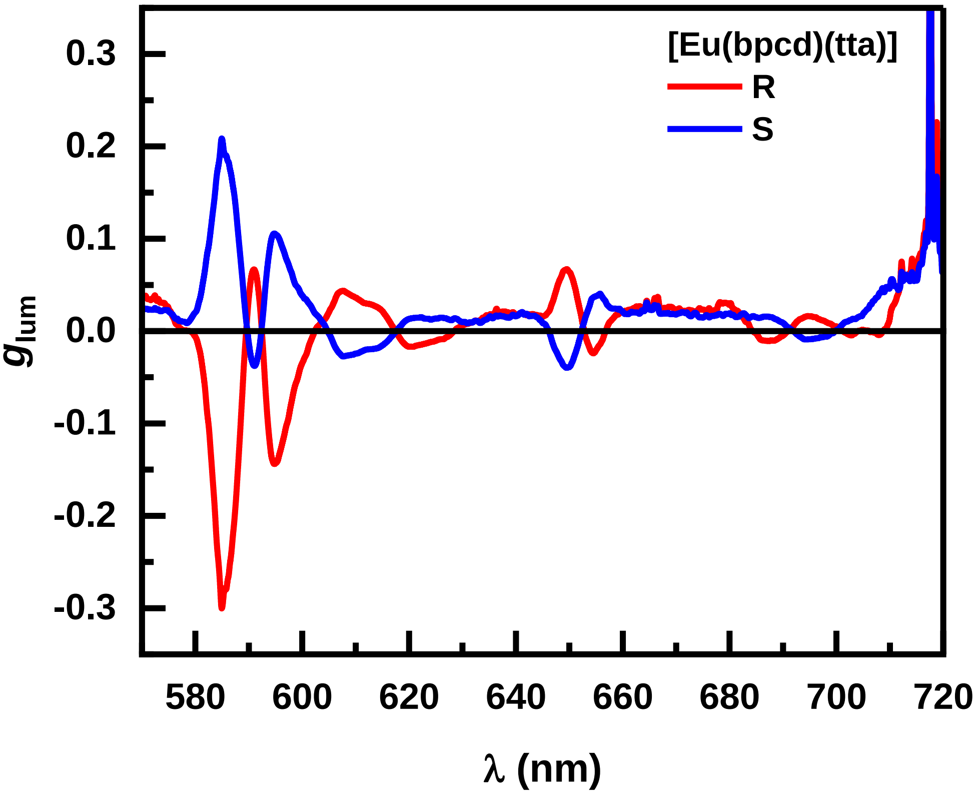

Figure S4. The g_{lum}-vs-wavelength plot for each enantiomer of [Eu(bpcd)(tta)].

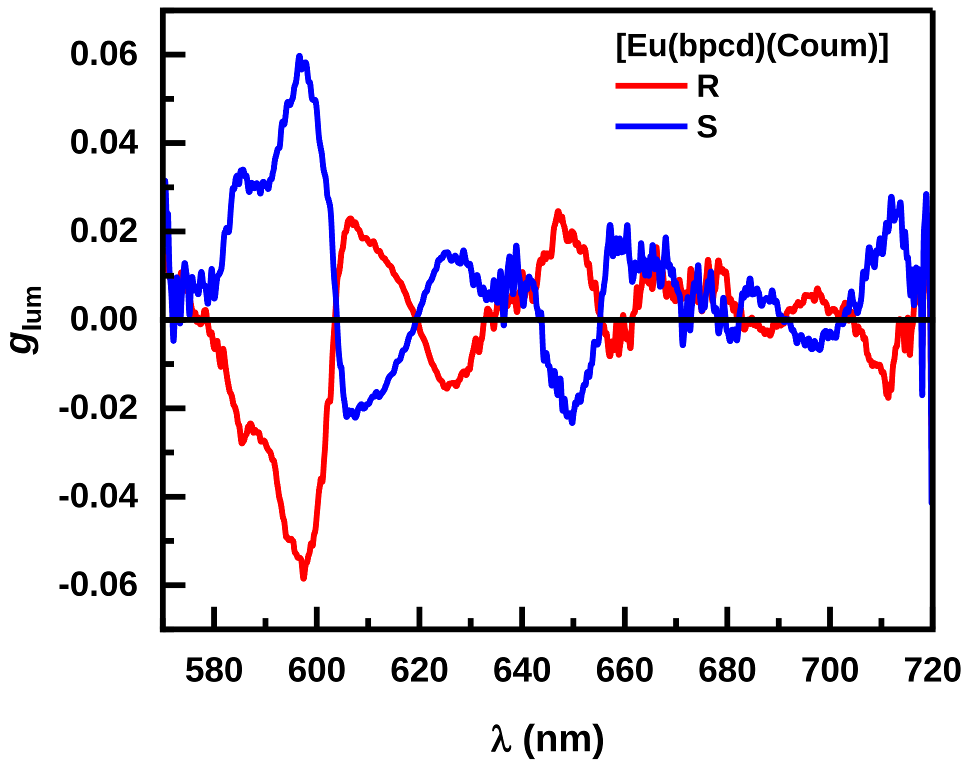

Figure S5. The g_{lum}-vs-wavelength plot for each enantiomer of [Eu(bpcd)(Coum)].

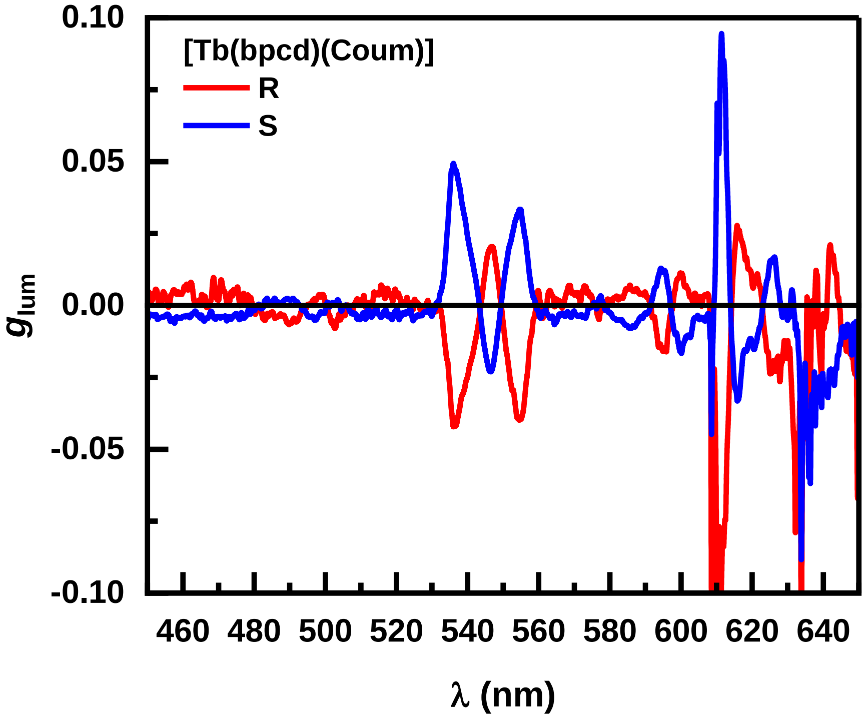

Figure S6. The g_{lum}-vs-wavelength plot for each enantiomer of [Tb(bpcd)(Coum)].

Figure S7. Kohn−Sham molecular orbitals composition of S_1 and T_1 states for [Y(bpcd)(Coum)]. Hydrogen atoms are omitted for clarity.

**SINGLETS**	**Occupied MO**		**Unoccupied MO**
trans-O,O<br>31514cm^{-1}	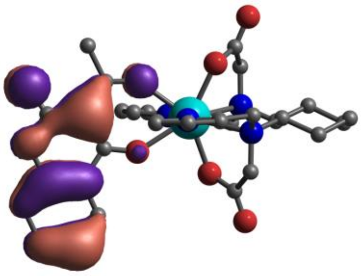<br>HOMO	→	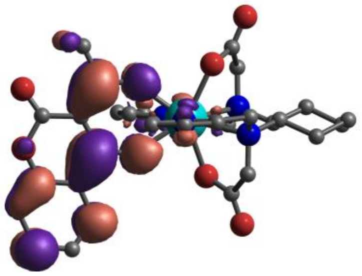<br>LUMO
trans-N,N<br>31865 cm^{-1}	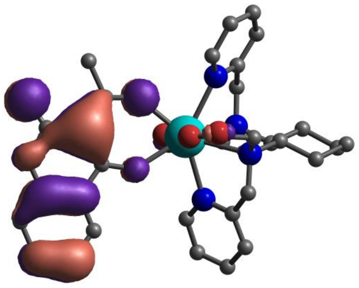<br>HOMO	→	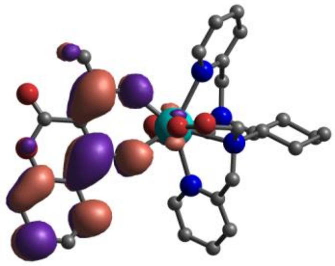<br>LUMO
cis-O,O,N,N<br>31769 cm^{-1}	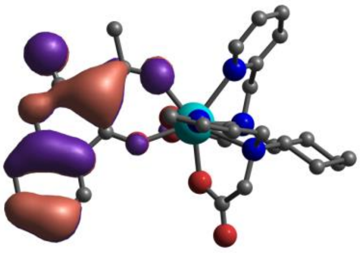<br>HOMO	→	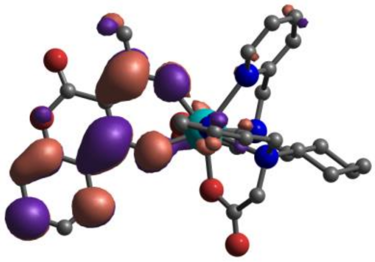<br>LUMO

TRIPLETS	Occupied MO		Unoccupied MO
trans-O,O<br>25636cm^{-1}	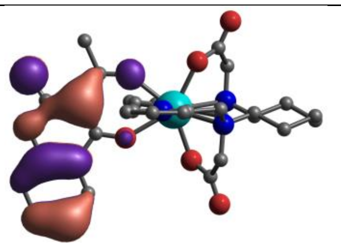<br>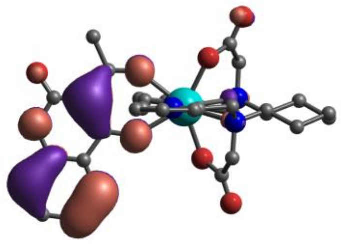<br>Homo 58%<br>Homo-1 28%	→	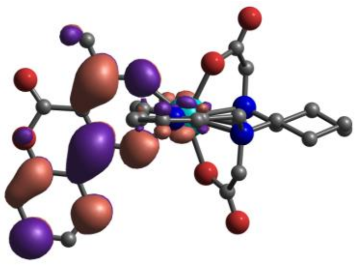<br>LUMO
trans-N,N<br>25606 cm^{-1}	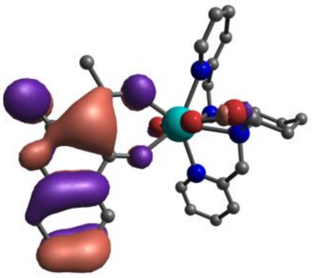<br>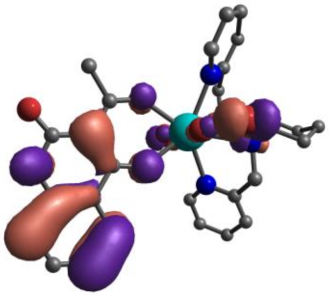<br>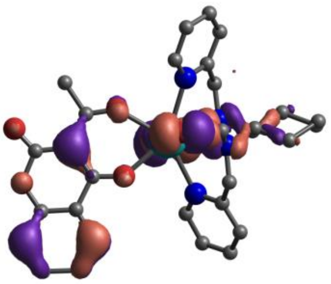<br>Homo 68%<br>Homo-1 11%<br>Homo-2 10%	→	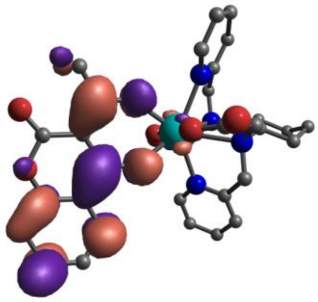<br>LUMO

cis-O,O,N,N

25612 cm^{-1}

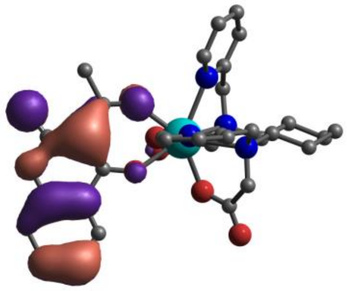

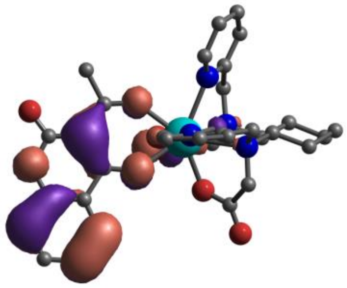

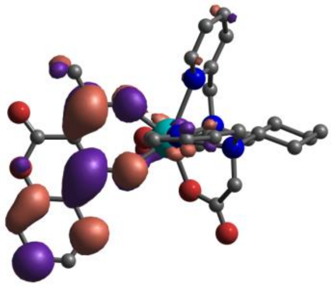

HOMO 63%

HOMO-1 18.3%

LUMO

Figure S8. Kohn−Sham molecular orbitals composition of S_1 and T_1 states for [Y(bpcd)(tta)]. H atoms are omitted for clarity.

SINGLETS	Occupied MO		Unoccupied MO
trans-O,O<br>29008 cm^{-1}	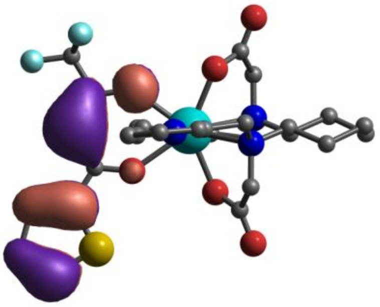<br>HOMO	→	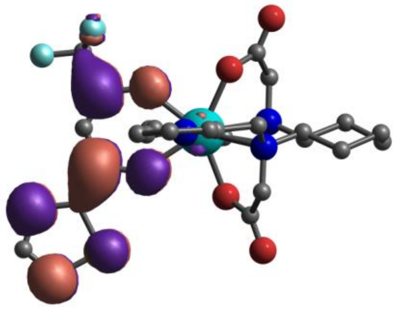<br>LUMO
trans-N,N<br>29138 cm^{-1}	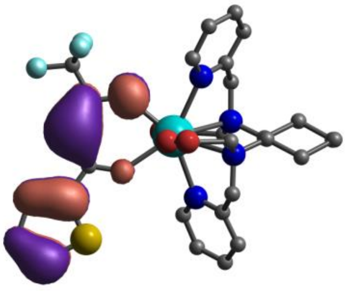		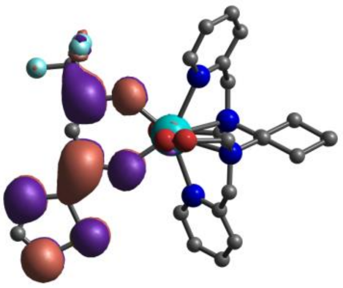

	HOMO	→	LUMO

cis-O,O,N,N<br>29122 cm^{-1}	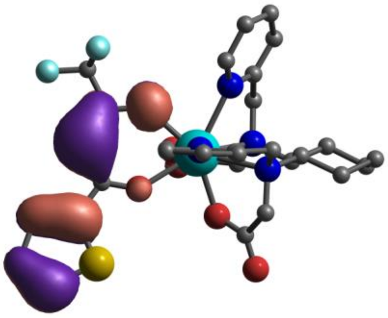<br>HOMO	→	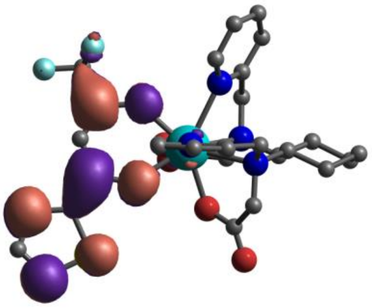<br>LUMO

**TRIPLETS**	**Occupied MO**		**Unoccupied MO**
trans-O,O<br>19640 cm^{-1}	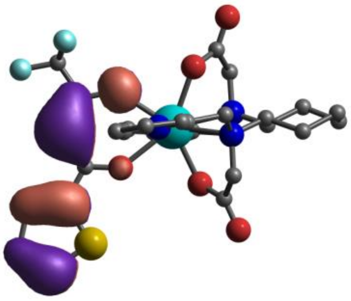<br>HOMO	→	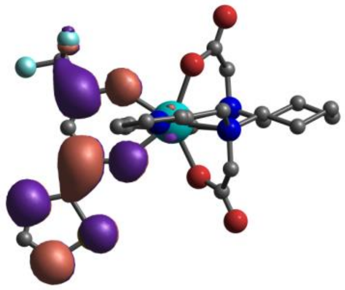<br>LUMO
trans-N,N<br>19582 cm^{-1}	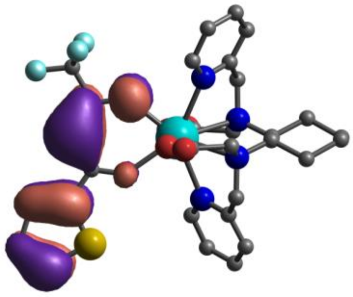<br>HOMO	→	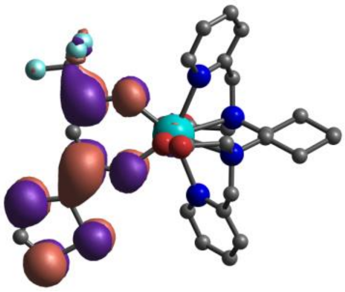<br>LUMO
cis-O,O,N,N<br>19631 cm^{-1}	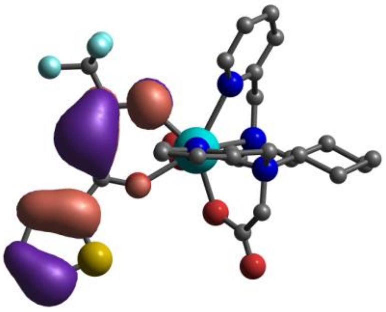<br>HOMO	→	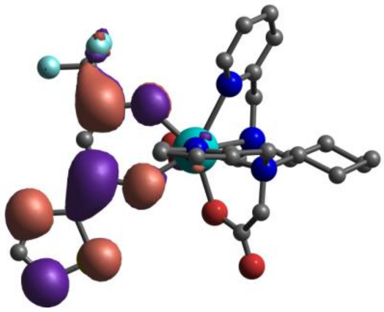<br>LUMO

Figure S9. Kohn−Sham molecular orbitals composition of S_1 and T_1 states for $[La(bpcd)(Coum)(H_2O)_n]$. Hydrogen atoms are omitted for clarity.

**SINGLETS**	**Occupied MO**	**Unoccupied MO**
trans-O,O (n =1)<br>32614 cm^{-1}	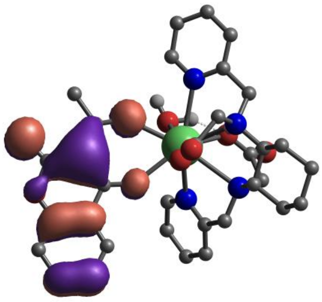<br>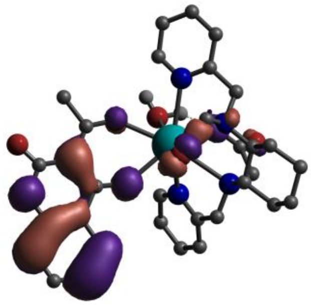<br>HOMO 87%<br>HOMO-2 10%	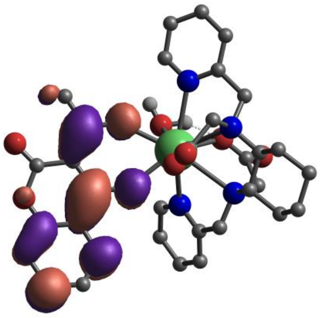<br>LUMO
trans-N,N (n = 2)<br>32552 cm^{-1}	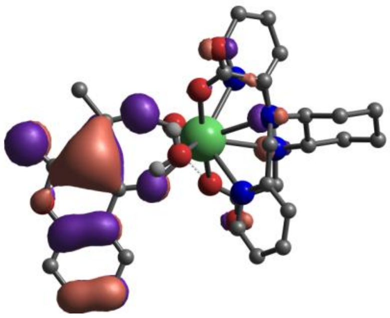<br>HOMO	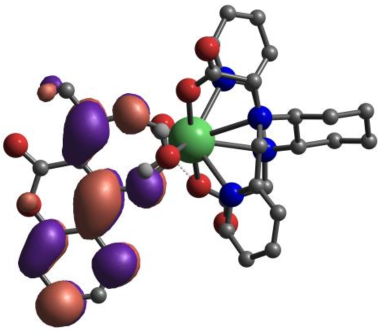<br>LUMO
cis-O,O,N,N (n=1)<br>32045 cm^{-1}	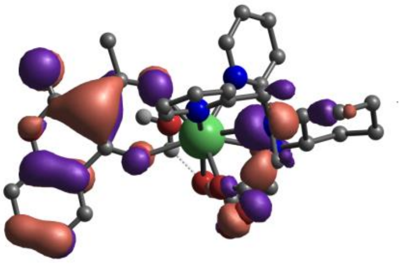	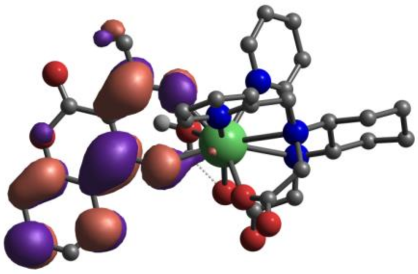

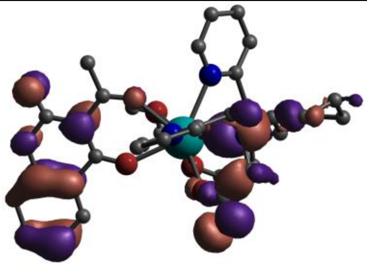

HOMO 65%

HOMO-1 27%

TRIPLETS	Occupied MO		Unoccupied MO
trans-O,O (n =1)<br>26033 cm^{-1}	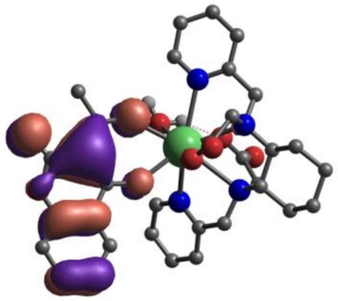<br>HOMO	→	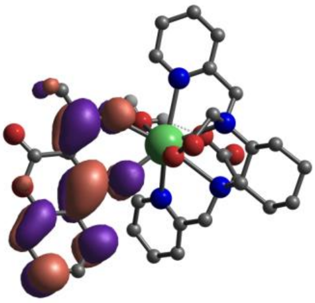<br>LUMO
trans-N,N(n =2)<br>25882cm^{-1}	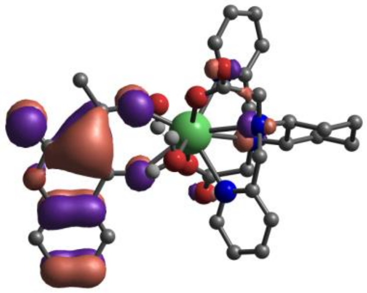<br>HOMO	→	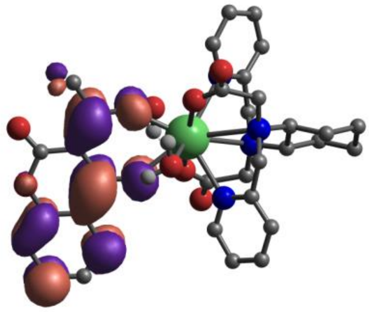<br>LUMO
cis-O,O,N,N(n =1)<br>25632 cm^{-1}	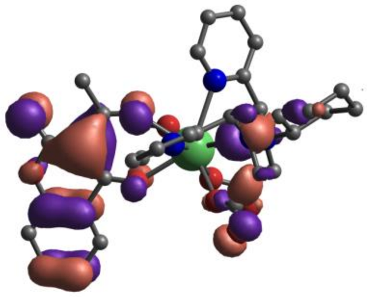	→	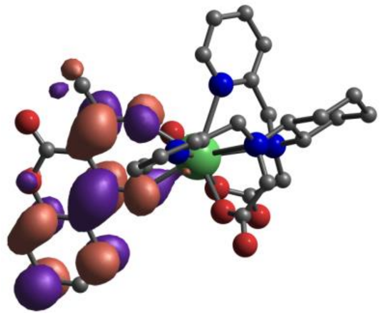

Figure S10. Kohn−Sham molecular orbitals composition of S_1 and T_1 states for [La(bpcd)(tta)$(H_2O)_n$]. Hydrogen atoms are omitted for clarity.

SINGLETS	Occupied MO		Unoccupied MO
trans-O,O (n =1)<br>29674 cm^{-1}	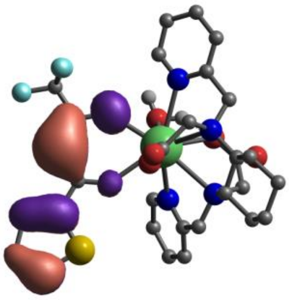<br>HOMO	→	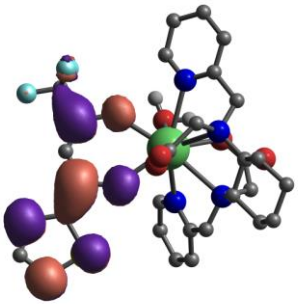<br>LUMO
trans-N,N(n =2)<br>29671 cm^{-1}	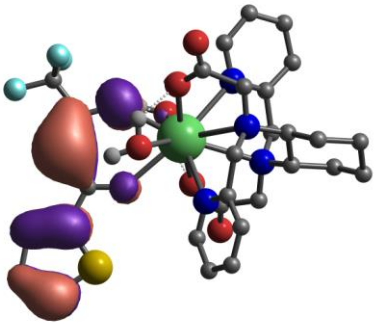<br>HOMO	→	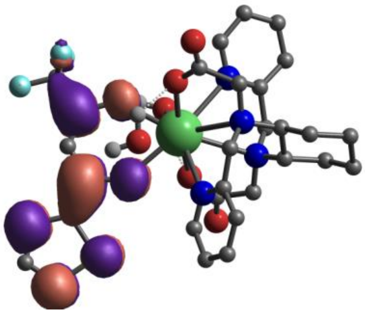<br>LUMO
cis-O,O,N,N(n =1)<br>29179 cm^{-1}	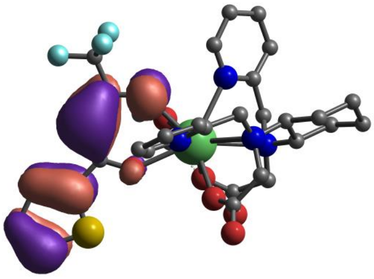<br>HOMO	→	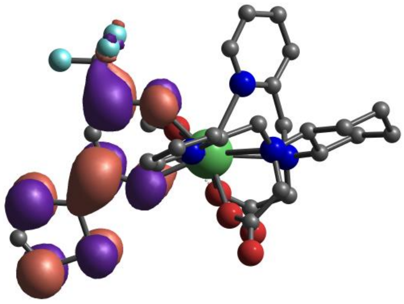<br>LUMO

TRIPLETS	Occupied MO	Unoccupied MO
trans-O,O (n =1)<br>20007 cm^{-1}	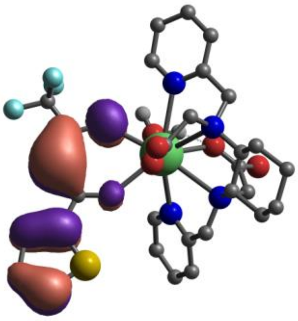<br>HOMO	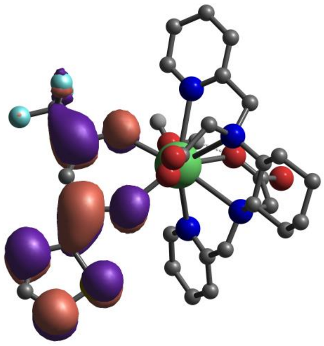<br>LUMO
trans-N,N(n =2)<br>20026 cm^{-1}	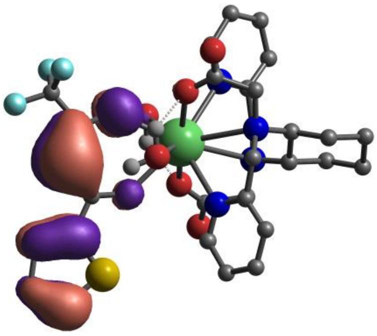<br>HOMO	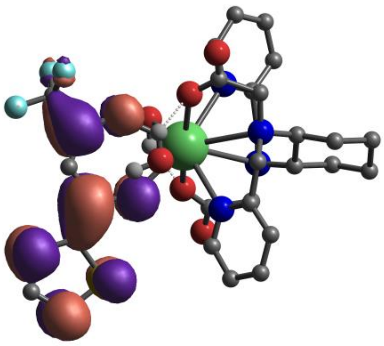<br>LUMO
cis-O,O,N,N(n =1)<br>19512 cm^{-1}	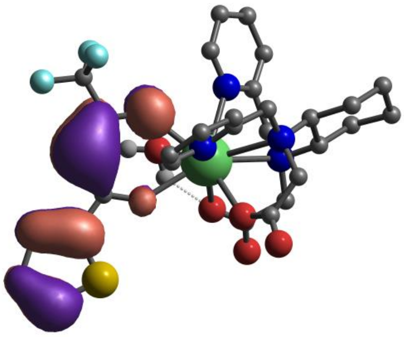<br>HOMO	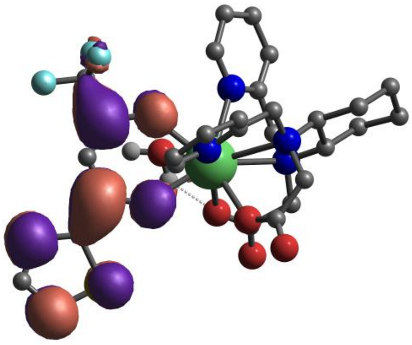<br>LUMO

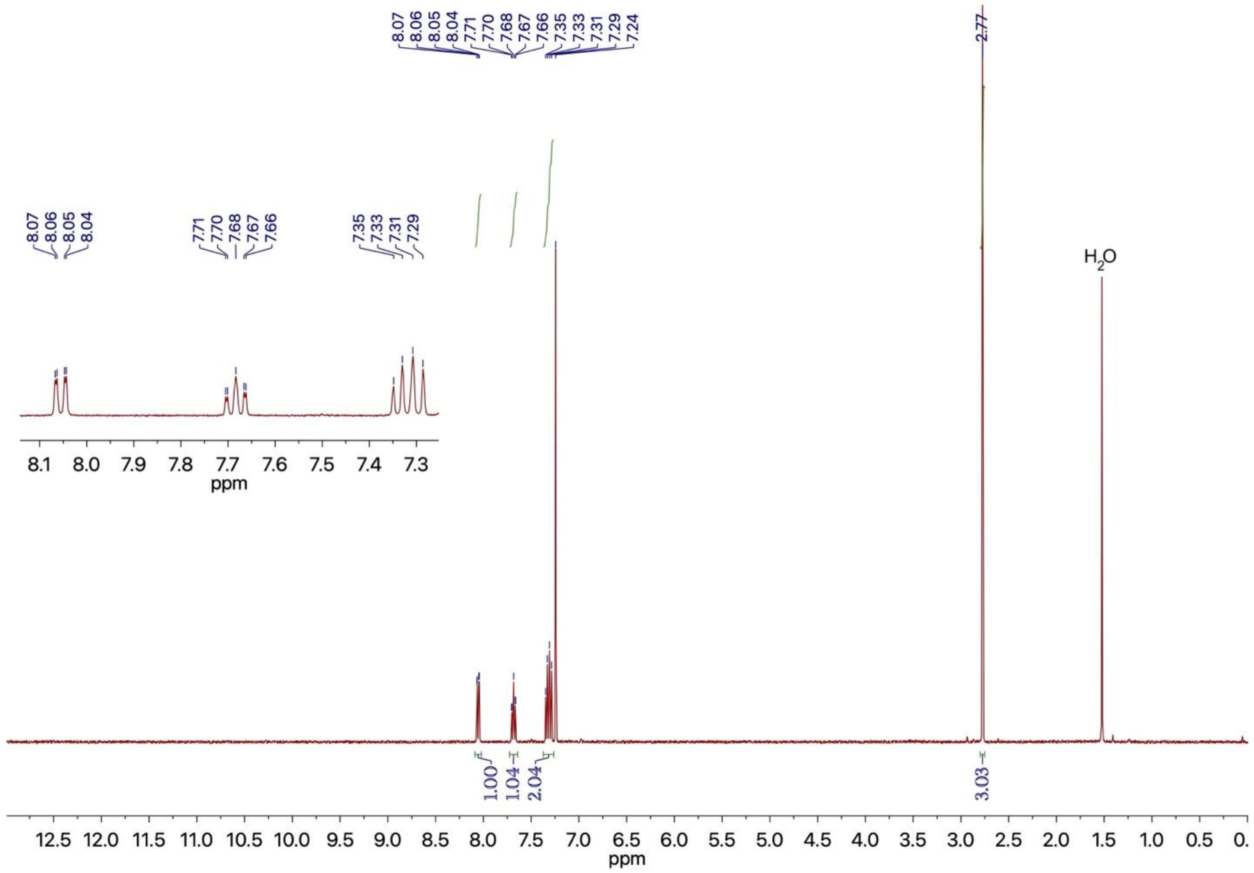

Figure S11. ^{1}H-NMR (400 MHz, $CDCl_3$) spectrum of 3-acetyl-4-hydroxy-2*H*-chromen-2-one.

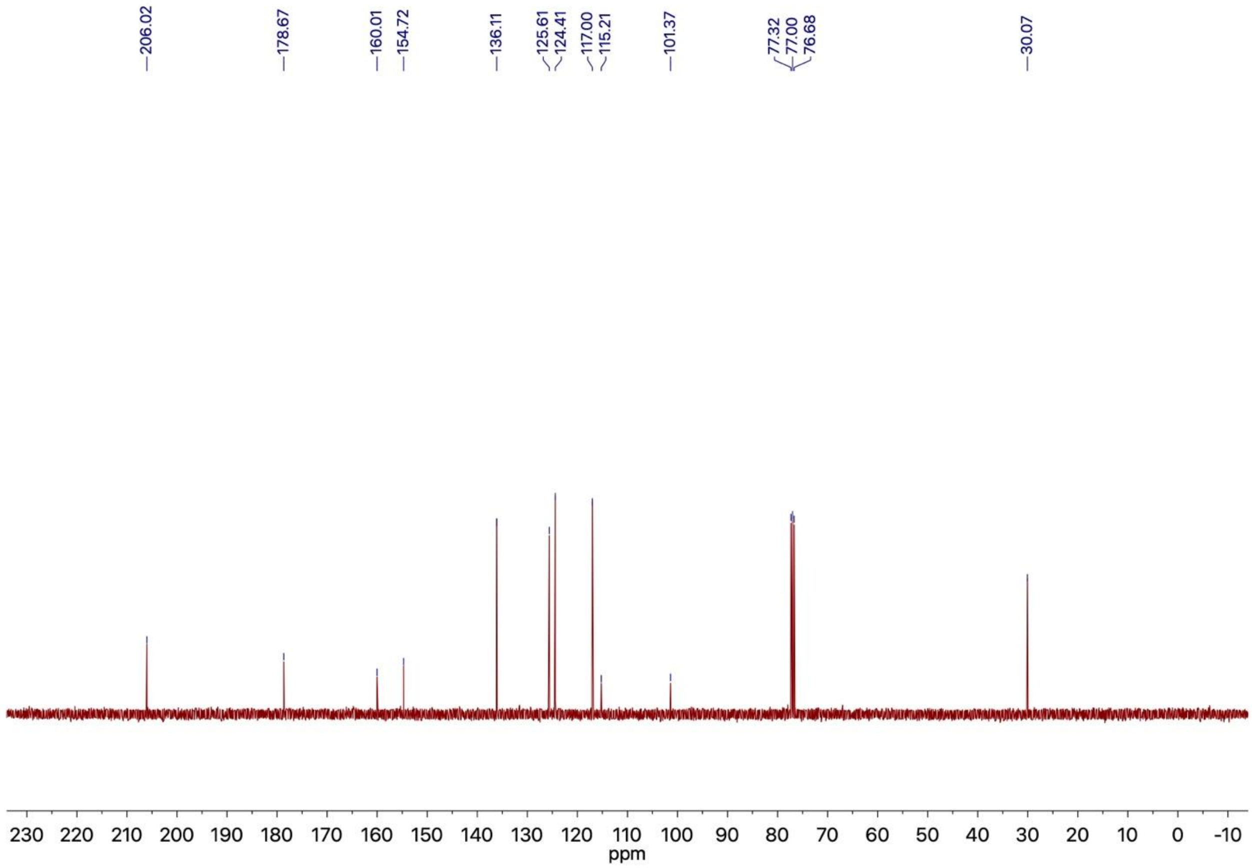

Figure S12. ^{13}C-NMR (100 MHz, $CDCl_3$) spectrum of 3-acetyl-4-hydroxy-2*H*-chromen-2-one.

References

[S1] L. Arrico, L. Di Bari, F. Zinna. Quantifying the Overall Efficiency of Circularly Polarized Emitters. *Chem. Eur. J.* **2021**, *27(9)*, 2920.
